# Utilization of machine learning for identifying symptom severity military-related PTSD subtypes and their biological correlates

**DOI:** 10.1038/s41398-021-01324-8

**Published:** 2021-04-20

**Authors:** Carole E. Siegel, Eugene M. Laska, Ziqiang Lin, Mu Xu, Duna Abu-Amara, Michelle K. Jeffers, Meng Qian, Nicholas Milton, Janine D. Flory, Rasha Hammamieh, Bernie J. Daigle, Aarti Gautam, Kelsey R. Dean, Victor I. Reus, Owen M. Wolkowitz, Synthia H. Mellon, Kerry J. Ressler, Rachel Yehuda, Kai Wang, Leroy Hood, Francis J. Doyle, Marti Jett, Charles R. Marmar

**Affiliations:** 1 Center for Alcohol Use Disorder and PTSD, Department of Psychiatry, New York University Grossman School of Medicine, New York, NY USA; 2 Division of Biostatistics, Department of Population Health, New York University Grossman School of Medicine, New York, NY USA; 3 Department of Psychiatry, James J. Peters VA Medical Center, Bronx, NY USA; 4 Department of Psychiatry, Icahn School of Medicine at Mount Sinai, New York, NY USA; 5 Military Readiness Systems Biology, Walter Reed Army Institute of Research, Silver Spring, MD USA; 6 Departments of Biological Sciences and Computer Science, The University of Memphis, Memphis, TN USA; 7 Department of Systems Biology, Harvard University, Cambridge, MA USA; 8 Harvard John A. Paulson School of Engineering and Applied Sciences, Harvard University, Cambridge, MA USA; 9 Department of Psychiatry, University of California, San Francisco, CA USA; 10 Department of Obstetrics, Gynecology, & Reproductive Sciences, University of California, San Francisco, CA USA; 11 Department of Psychiatry, McLean Hospital, Belmont, MA USA; 12 Institute for Systems Biology, Seattle, WA USA

**Keywords:** Diagnostic markers, Scientific community

## Abstract

We sought to find clinical subtypes of posttraumatic stress disorder (PTSD) in veterans 6–10 years post-trauma exposure based on current symptom assessments and to examine whether blood biomarkers could differentiate them. Samples were males deployed to Iraq and Afghanistan studied by the PTSD Systems Biology Consortium: a discovery sample of 74 PTSD cases and 71 healthy controls (HC), and a validation sample of 26 PTSD cases and 36 HC. A machine learning method, random forests (RF), in conjunction with a clustering method, partitioning around medoids, were used to identify subtypes derived from 16 self-report and clinician assessment scales, including the clinician-administered PTSD scale for DSM-IV (CAPS). Two subtypes were identified, designated S1 and S2, differing on mean current CAPS total scores: S2 = 75.6 (sd 14.6) and S1 = 54.3 (sd 6.6). S2 had greater symptom severity scores than both S1 and HC on all scale items. The mean first principal component score derived from clinical summary scales was three times higher in S2 than in S1. Distinct RFs were grown to classify S1 and S2 vs. HCs and vs. each other on multi-omic blood markers feature classes of current medical comorbidities, neurocognitive functioning, demographics, pre-military trauma, and psychiatric history. Among these classes, in each RF intergroup comparison of S1, S2, and HC, multi-omic biomarkers yielded the highest AUC-ROCs (0.819–0.922); other classes added little to further discrimination of the subtypes. Among the top five biomarkers in each of these RFs were methylation, micro RNA, and lactate markers, suggesting their biological role in symptom severity.

## Introduction

Distinct subtypes of posttraumatic stress disorder (PTSD), a heterogeneous disorder, have been sought to isolate differentiating clinical features and their biological mechanisms so that recommendations can be made for more precise treatments and prognostic indicators can be identified. A natural starting point for developing clinical subtypes is derived from the four sets of symptom criteria for PTSD defined in the diagnostic statistical manual (DSM-5)^1,2^: intrusion, avoidance, negative alterations in cognitions and mood, and alterations in arousal and reactivity. Subtype suggestions have been made based on externalization features^3^, and for a dissociative subtype that includes depersonalization and derealization symptoms and delayed onset^4,5^. Other symptoms that commonly co-occur with PTSD, including anxiety and depressive symptoms, have also been considered in the search for PTSD subtypes^6^. Clusters of distinct clinical symptoms are desirable for defining subtypes, but several subtyping studies at best have led to subgroups distinguished on symptom severity and comorbidities^7^. While biological correlates of PTSD including multi-omic blood biomarkers^8^, cortisol^9^, neurocognitive markers^10^, neuroimaging markers^11,12^, and voice markers^13^ have been studied, to date only a few studies have shown their value for characterizing subtypes. Among these, neurocognitive functioning has been shown to differentiate clinically defined severity subtypes^6^. The dissociative subtype of PTSD is associated with altered resting-state functional connectivity of the amygdala^11^ and altered subcortical white matter connectivity^12^. Zhang et al.^14^ demonstrated that electroencephalography (EEG) functional connectivity defined subtypes for PTSD and major depressive disorder (MDD) predict differential treatment response when comparing psychotherapy to placebo for PTSD, and antidepressant medication versus placebo for MDD. Notably, epigenetic markers have been used to define PTSD subtypes, which are then shown to differ on clinical characteristics^15^, a reversal of the usual approach.

Analytic methods used to identify subtypes have evolved from statistical clustering approaches to modern methods of machine learning that are essentially unsupervised searches for patterns involving between persons’ distance measures. The most common statistical method used in prior studies of the heterogeneity in PTSD symptom presentations is latent profile analysis (LPA)^5,6,16,17^ in which the probability of being in a latent class is modeled. The probability is assumed to be represented by a weighted mixture of normally distributed random variables in which each represents a subtype and the weights are the probabilities of subtype membership for a given feature profile. Normality assumptions, difficulty in parameter estimation, and limits on the number of variables that can be considered reduce the usefulness of this statistical approach. In our study, we used the machine learning method of random forests (RF)^18^ to obtain a distance measure between subjects in conjunction with an unsupervised statistical clustering method, partitioning around medoids (PAM)^19^, to identify subtypes. This is a novel approach that quantifies the distance between subjects as small if they frequently fall in the same terminal nodes of the RF trees grown to classify the disorder versus healthy controls (HC). RF are also used in our report to identify biological correlates of the subtypes. Several advantages accrue to the use of RF most germane here is the ability to include large sets of both binary and continuous features even with modest sample sizes, and to provide a distance metric for clustering that is related to the purpose of the clusters. Further, RF are developed with internal validation procedures achieved through out-of-bag sampling^20^ in which numerous repetitions of random bootstrap samples used in training models are evaluated on the left-out samples to obtain robust estimates of the AUC of the RF ROC and its modeling errors. Sophisticated variable reduction methods are available such as “shaving”^21^ based on measures of a feature’s importance to the classifier^22^ and enable a reduction in the number of features considered.

**Fig. 1:**
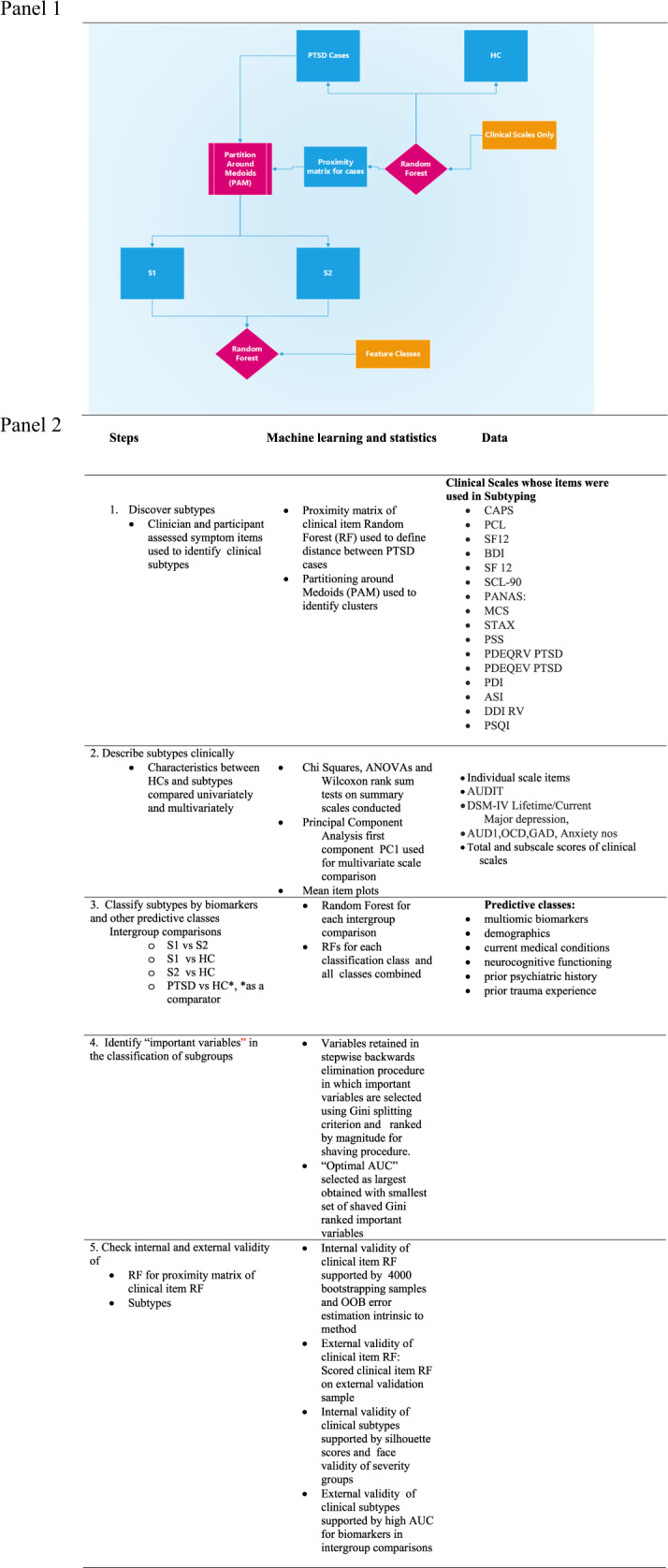
Flow chart of analytic steps and specific procedures. Panel 1: Flow chart of analytic steps. Panel 2: Specific procedures.

The goal of the current study is to discover PTSD subtypes that are homogeneous in clinical symptoms and to determine whether multi-omic blood biomarkers can differentiate them. To identify subtypes, we considered a large array of clinical symptom items captured in 16 validated self-report and clinical assessment scales (see Fig. 1 and Table S1) that are commonly used in practice to assess PTSD and its comorbidities in clinical research settings. The distance between subjects is captured by the proximity matrix of an RF grown to classify cases vs HC with these clinical symptoms. To establish the comparative importance of multi-omic blood biomarkers in contrast with other candidate predictor classes of subtypes, we also examined whether five additional feature classes improved classification. The novelty of our work lies in the large number of clinical items considered in subtyping, the use of a proximity matrix from an RF in a clustering algorithm to identify the symptom severity subtypes, and the separate validation of the subtypes with a large class of multi-omic markers that previously have been demonstrated by our group to differentiate PTSD from HC^8^.

While a best-practice procedure for the external validation of subtypes has not yet been described in the psychometric or statistical literature, the logic and flow of the present work have been informed by the criteria described by Dalenberg et al.^23^. These authors propose that to be useful subtypes must be (1) clinically relevant, (2) reliably measured, and (3) have different biological mechanisms. We address each of these criteria as follows: (1) clinical relevance is attested to by the intuitive face validity of the identified clusters, (2) reliable measurement by the use of only items from reliable and well-validated assessment scales as defining features, and (3) the different biological mechanisms of subtypes shown by the ability of a parsimonious set of biomarkers to accurately classify subjects into the clinical subtypes.

## Methods

### Background

The discovery and validation samples utilized for subtyping are male Iraq and Afghanistan veterans, a group documented to have an increased risk for PTSD. The lifetime prevalence of PTSD in US veterans of the Vietnam War and subsequent conflicts, including the Iraq and Afghanistan Wars, is estimated to be between 10.1% and 30.9%^24–26^. We studied participants from the PTSD system biology consortium (SBC)^8^, a collaboration of the US Army and multiple academic centers enrolling subjects, identifying biological markers, and advancing diagnostics for high throughput clinical screening to identify military service-related PTSD. Comprehensive biological, clinical, and neurocognitive data on Iraq and Afghanistan male veterans exposed to military trauma were assessed 6–10 years post-deployment. Participants were evaluated with the clinician-administered PTSD scale for DSM-IV (CAPS)^1^ to determine if they met diagnostic criteria, the structured clinical interview for DSM–IV (SCID)^27^ to assess other psychiatric disorders, and the peritraumatic dissociative experiences questionnaire^28^, a rater measure of peritraumatic dissociation, self-report measures of symptoms of PTSD and associated co-morbidities and measures of neurocognitive functioning. Historical information was collected on adverse childhood events and pre-military trauma exposure. Blood was drawn yielding over one million biomarkers including GWAS, DNA methylation, miRNAs, metabolomics, proteomics, small molecules, endocrine markers, routine clinical lab panels, and biometric/physiological markers. A mixed-method approach of analytic and qualitative methods labeled “wisdom of crowds” was used to reduce features into 343 unique candidates and include, as reported in our paper^8^, COMBINER^29^, polygenic risk^30,31^, as well as traditional SVM-RFE, RF, and other classification algorithms and feature selection approach, including *p*-value, *q*-value, and fold-change filtering. From the 343 features, 28 were down-selected to be included in an RF classifier of previously deployed Iraq and Afghanistan veterans with military service-related PTSD cases and HC. The PTSD SBC sample was comprised of 166 male veterans, 83 who met DSM-IV diagnostic criteria for a current diagnosis of PTSD with a CAPS total score ≥40 (cases), and 83 who were HC with a CAPS total score ≤20.

### Discovery sample

Our discovery sample is a subset of the 166 male Iraq and Afghanistan veterans drawn from our PTSD SBC sample^8^. Besides the above-specified cut-points for CAPs (inclusion criteria), other inclusion/exclusion criteria included stable on medications in the prior month, no prominent suicidality in the past three months, no psychotic or bipolar disorders, no severe drug use disorder in the past year, no open head injury and no major medical illness or neurological conditions. The HC were selected similar in age and ethnicity to the cases. The discovery sample size was reduced from 166 to 145 because some missing data could not be validly imputed; the resulting discovery sample size for the current study was 74 cases and 71 HC.

### Validation sample

An independent validation sample of 62 male Iraq and Afghanistan veterans, 26 PTSD cases and 36 HC, also drawn from the earlier SBC study^8^ with similar characteristics to the discovery sample, was used in the current study to validate the RF that generated the distance metric for the clustering algorithm used to find subtypes. Table 1 displays and compares background characteristics of the discovery and validation samples.

**Table 1:** Comparison of HC and PTSD cases in discovery (D) and validation (V) samples.

	Discovery (D)		Validation (V)		D vs. V HC	D vs. V PTSD
	HC *n* = 71	PTSD *n* = 74	HC *n* = 36	PTSD *n* = 26	*p*-value	*p*-value
Age (years, Mean (sd))	32.58 (7.85)	32.50 (7.33)	33.83 (9.22)	36.83 (10.24)	0.463	0.022
Ethnicity (*n*, %)						
Hispanic	23 (32.39%)	33 (44.59%)	6 (16.67%)	11 (42.31%)	0.079	0.211
Non-hispanic Asian	5 (7.04%)	1 (1.35%)	6 (16.67%)	3 (11.54%)		
Non-hispanic black	17 (23.94%)	20 (27.03%)	6 (16.67%)	5 (19.23%)		
Non-hispanic white	23 (32.39%)	19 (25.68%)	18 (50.00%)	7 (26.92%)		
Non-hispanic other	3 (4.23%)	1 (1.35%)	0 (0.00%)	0 (0.00%)		
Education (*n*, %)						
Less than 12th grade	0 (0.00%)	1 (1.35%)	1 (2.78%)	1 (3.85%)	0.464	0.543
HS diploma or GED	13 (18.31%)	27 (36.49%)	10 (27.78%)	10 (38.46%)		
2 years college, AA degree	19 (26.76%)	22 (29.73%)	8 (22.22%)	7 (26.92%)		
4 years college, BA degree	27 (38.03%)	21 (28.38%)	10 (27.78%)	5 (19.23%)		
Masters degree	11 (15.49%)	3 (4.05%)	7 (19.44%)	3 (11.54%)		
Doctoral degree	1 (1.41%)	0 (0.00%)	0 (0.00%)	0 (0.00%)		
Body mass index (mean (sd))	28.11 (4.36)	30.13 (5.23)	25.36 (8.38)	28.32 (8.76)		
Cholesterol (mean (sd))						
HDL cholesterol	50.30 (13.39)	47.81 (11.67)	52.35 (11.91)	46.85 (14.70)	0.441	0.740
LDL cholesterol	99.55 (25.50)	109.51 (32.12)	100.26 (27.71)	111.28 (31.50)	0.894	0.810
PTSD severity, Total CAPS (Mean (sd))	3.37 (4.66)	68.12 (16.02)	3.97 (5.46)	67.15 (19.37)	0.550	0.803
Major depressive disorder (current depression) (*n*, %)	0 (0.00%)	41 (55.41%)	3 (8.33%)	9 (34.62%)	0.060	0.111
Current alcohol use % none (*n*, %)	10 (14.08%)	20 (27.03%)	5 (13.89%)	2 (7.69%)	0.422	0.235

## Variables

### Clinical items for finding subtypes of PTSD

Figure 1 and Table S1 list the 16 clinical scales whose individual items were used to subtype cases and include the CAPS, the PDEQ (a rater administered peritraumatic dissociative experiences scale), and other commonly used reliable and validated self-report symptom scales.

### Feature classes for describing and classifying symptom severity subtypes

The multi-omic blood biomarkers used for classifying the identified clinical PTSD subtypes included GWAS, DNA methylation, miRNAs, metabolomics, proteomics, small molecules, endocrine, routine clinical labs, and biometric/physiological markers. They are the same as 343 selected in our earlier study for classifying PTSD cases and HC^8^, with the exception of GABR which was removed because of extreme outlier values. The markers are displayed in Table S2 and the number of markers within a class in Table S3. Five additional feature classes were included to determine their value in classification: current medical co-morbidities, current neurocognitive functioning, demographics, past psychiatric history, and pre**-**military trauma. None of these feature classes were incorporated into the primary search for clinical subtypes as they are not easily collected in most clinical settings (e.g., neurocognitive functioning), are not current mental state assessments (e.g., past psychiatric history, pre-military trauma), or had category frequencies in the sample that were non-representative of the target population (e.g., demographics) used in forming the subtypes but are considered for their ability to discriminate the clinical subtypes. To further describe the sample and derived subtypes, current SCID diagnoses of alcohol and depressive psychiatric disorder were also collected.

## Data analytic methods

Figure 1 summarizes the analytic steps of the study procedures.

### Random forests

The machine learning method used was RF, an ensemble method that combines independent decision trees whose terminal nodes determine class membership^18^. A final vote summarizes the individual tree results generating a probability of membership in a class. Each tree in an RF uses an internal validation method, a bootstrap method to select a sample from the dataset to train the decision tree, and the remaining sample (out-of-bag, OOB) to estimate the prediction error^20^. This can be repeated multiple times as specified by the user. Varying the probability threshold-level defining membership in a class generates a receiver operating curve (ROC) and its area under the curve (AUC-ROC), which is used to measure classification accuracy. The importance of each feature to the RF, the mean reduction in Gini index^22^, is measured by the average reduction obtained in the ambiguity of classification at a node when it is used as a splitting variable in comparison to ambiguity at the two descendant nodes of the split. Shaving eliminates less important variables to classification^21^. The least important features are first eliminated, the RF rerun with the reduced set, its AUC computed and the process continued until an optimal AUC is selected as the largest value obtained with the smallest set of unshaved variables. The programs were run using R software^32^ and specialized source coding for modeling and subsequent clustering have been placed in GitHub^33^.

### Clustering method to find subtypes based on clinical symptoms

Subtypes of PTSD cases were obtained using the clustering program, PAM^19^. The distance between subjects, a required input to any clustering algorithm, was obtained by a novel use of the proximity matrix of an RF to classify cases versus HC. An RF parses subjects based on feature splits ending in a terminal node in which persons have the same pattern of feature interactions. The proximity between two PTSD cases is defined based on the frequency across the decision trees of the RF of being in the same terminal node. For the PAM algorithm, the measure of distance between cases was one minus the entries in the resulting proximity matrix. Because the discovery and validation sample sizes were not large, we restricted the number of clusters to two, and label the resulting clusters S1 and S2.

### Classifying subtypes with biomarkers and other feature classes

To estimate how well biomarkers classified subjects into subtypes, three RFs classifier models were grown with multiple feature classes: S1 vs. HC, S2 vs. HC, S2 vs. S1, and an RF to classify all cases vs HC for comparative purposes. For each of these, six separate RFs were grown using the six supplementary feature classes, and their AUC-ROCs compared to assess the extent to which they improved classification over that from blood biomarkers alone (see Table S4). Additional RF pooling of all classes was also grown.

### Canonical correlation

We used canonical correlations^34^ to examine whether the variables we found to be “important” in our RF distinguishing cases and controls were correlated with the variables we found in our earlier classifier for PTSD cases and controls^8^. To compare the relationship between two sets of features, *X*_1_, *X*_2_,…*X*_u_ and *Y*_1_, *Y*_2_, …*Y*_*v*_, canonical-correlation analysis was used to find the linear combination of the *X* variables and the linear combination of the *Y* variables that has the maximum correlation with each other. The pair of linear combinations of the *X*’s and *Y*’s are called the first canonical variables. In a second step, a similar maximization procedure seeks to find the linear combination of the *X* variables and the linear combination of the *Y* variables that maximizes their correlation subject to the constraint that they are uncorrelated with the first pair of canonical variables. The method continues until the canonical correlations are too small to be of interest. The maximum number possible is the smaller of *u* and *v*. We report on the first three canonical correlations obtained from comparing the important biomarkers from our earlier and current study.

### Validation approaches

Internal validation of any of the RFs grown for this study was intrinsic to the bootstrap method used in which we employed 4000 bootstrap samples to grow the trees, and estimated errors from the out-of-bag samples. For external validation of the RF used for clustering, it was scored with the independent but demographically similar validation sample of 62 male veterans who had been assessed on the same clinical scales. Table 1 displays the results of a comparison of the characteristics of the discovery to the validation sample.

Internal validation of the PAM clusters was measured by silhouette scores^35^ that calculate the extent to which members in a cluster are close to each other and distant from members in other clusters (see Fig. S1a). Multidimensional scaling was used to visualize the clusters via a diffusion map^36^ (see Fig. S1b). Face validity of the derived subtypes was appraised by determining whether differences in the item scores between the individuals in the subtypes were statistically different and clinically interpretable. Figure S2 displays plots of the mean item clinical scores among groups. Statistical differences in the total scale and subset scores of the clinical scales between S1, S2, and HC were tested with an ANOVA; and between: S1 vs. HC, S2 vs. HC, and S2 vs. S1 with Wilcoxon rank-sum tests. Significance is reported based on a family-wise error rate, (*p* < 0.0001). Results are displayed in Table 2. Totals and subscales of the clinical scores were also multivariately compared for the first principal component (PC1) (see Table S5) of a principal component analysis (see the first entry in Table 2).

**Table 2:** Clinical assessments of HCs, S1, S2: ANOVAs and *t*-test comparisons of mean (sd) scores for total and subscale scores and for principal component 1 of a multiscale PCA analysis.

Variables	HCs	S1	S2	*p* ^*^values			
				S1 vs. S2 vs. HC	S1 vs. HC	S2 vs. HC	S2 vs. S1
PC1	3.97 (1.75)	1.57 (2.65)	5.02 (3.10)	<0.0001	<0.0001	<0.0001	<0.0001
CAPSTOT	3.37 (4.66)	54.31 (6.61)	75.60 (14.58)	<0.0001	<0.0001	<0.0001	<0.0001
PCLSCORE	25.20 (8.27)	53.69 (11.03)	64.81 (10.98)	<0.0001	<0.0001	<0.0001	<0.0001
SCLSOM	0.22 (0.27)	0.97 (0.63)	1.47 (0.82)	<0.0001	<0.0001	<0.0001	0.008*
SCLOC	0.56 (0.67)	1.77 (0.90)	2.30 (0.82)	<0.0001	<0.0001	<0.0001	0.013*
SCLINT	0.31 (0.45)	1.21 (0.83)	1.85 (0.92)	<0.0001	<0.0001	<0.0001	0.004*
SCLDEP	0.32 (0.41)	1.40 (0.77)	2.09 (0.78)	<0.0001	<0.0001	<0.0001	0.001*
SCLANX	0.16 (0.24)	1.07 (0.71)	1.89 (0.85)	<0.0001	<0.0001	<0.0001	<0.0001
SCLHOS	0.25 (0.31)	1.27 (1.06)	2.08 (0.96)	<0.0001	<0.0001	<0.0001	0.001*
SCLPHOB	0.09 (0.17)	0.88 (0.64)	1.74 (0.81)	<0.0001	<0.0001	<0.0001	<0.0001
SCLPAR	0.32 (0.40)	1.37 (0.90)	1.78 (1.04)	<0.0001	<0.0001	<0.0001	0.097*
SCLPSY	0.18 (0.28)	0.76 (0.62)	1.34 (0.82)	<0.0001	<0.0001	<0.0001	0.002*
SCLGSI	0.280 (0.30)	1.23 (0.61)	1.86 (0.72)	<0.0001	<0.0001	<0.0001	<0.0001
SCLPST	17.39 (15.46)	53.19 (20.92)	67.85 (14.62)	<0.0001	<0.0001	<0.0001	0.001*
SCLPSDI	1.30 (0.31)	2.04 (0.43)	2.42 (0.64)	<0.0001	<0.0001	<0.0001	0.008*
PSQI	5.87 (3.58)	11.73 (3.37)	13.88 (3.13)	<0.0001	<0.0001	<0.0001	0.008*
BDI_total	5.37 (5.42)	17.50 (8.65)	28.75 (9.99)	<0.0001	<0.0001	<0.0001	<0.0001
PANAS_PA	34.46 (8.6)	27.92 (6.70)	23.88 (7.27)	<0.0001	0.001	<0.0001	0.022*
PANAS_NA	15.06 (4.37)	23.00 (7.25)	31.65 (7.90)	<0.0001	<0.0001	<0.0001	<0.0001
MCS	63.20 (12.81)	106.42 (15.38)	123.79 (19.17)	<0.0001	<0.0001	<0.0001	<0.0001
STAX	15.66 (1.45)	21.0 8 (7.79)	26.98 (12.30)	<0.0001	<0.0001	<0.0001	0.03*
PSS	1.79 (0.58)	2.73 (0.72)	3.22 (0.57)	<0.0001	<0.0001	<0.0001	0.002*
PDEQRV	1.23 (0.25)	1.72 (0.50)	1.87 (0.43)	<0.0001	<0.0001	<0.0001	0.198*
PDEQEV	0.42 (0.58)	1.54 (1.050)	2.12 (0.93)	<0.0001	<0.0001	<0.0001	0.016*
PDI rv	1.08 (0.60)	1.86 (0.92)	2.24 (0.64)	<0.0001	<0.0001	<0.0001	0.041*
PDI ev	0.86 (0.58)	1.82 (0.73)	2.32 (0.79)	<0.0001	<0.0001	<0.0001	0.01*
ASI	11.49 (9.81)	16.89 (10.03)	28.98 (14.07)	<0.0001	0.019*	<0.0001	<0.0001
sumERS	35.01 (7.81)	26.42 (6.26)	23.33 (8.08)	<0.0001	<0.0001	<0.0001	0.095*
PF_T	52.96 (7.76)	48.54 (9.07)	45.19 (12.55)	<0.0001	0.02*	<0.0001	0.235*
RP_T	53.61 (7.40)	47.43 (8.87)	43.07 (12.83)	<0.0001	0.001*	<0.0001	0.127*
BP_T	51.99 (9.25)	45.68 (10.67)	41.52 (13.28)	<0.0001	0.005*	<0.0001	0.173*
GH_T	48.63 (9.89)	41.67 (10.26)	37.28 (11.97)	<0.0001	0.003*	<0.0001	0.118*
VT_T	52.29 (9.13)	41.17 (9.84)	38.53 (9.48)	<0.0001	<0.0001	<0.0001	0.262*
RE_T	49.86 (9.89)	40.38 (10.25)	30.80 (13.30)	<0.0001	<0.0001	<0.0001	0.002*
SF_T	51.88 (9.17)	38.31 (9.48)	30.69 (11.20)	<0.0001	<0.0001	<0.0001	0.004*
MH_T	51.41 (9.20)	39.92 (9.04)	31.01 (9.90)	<0.0001	<0.0001	<0.0001	<0.0001

*Not significant using Bonferroni corrected *p* value, *p* < 0.0001.

However, to our knowledge, there are no available methods to externally validate the clusters themselves, as the ground truth, i.e., the “true” clusters in a validation sample are not known. Further, our external validation sample contained only 26 PTSD cases which, even if we knew the truth, would generate even smaller samples in the subtypes for any meaningful statistical testing. The ability to accurately classify persons into the PTSD clinical subtypes based on the blood biomarkers, a form of concurrent validity, was considered to provide external validation of the subtypes and by Dalenberg’s criteria of meaningfulness of clinical subtypes^23^ that they are distinguishable by distinct biological mechanisms.

## Results

### Identifying PTSD subtypes in the discovery sample

Two clinical subtypes of PTSD cases were found with PAM: the first, designated as S1, comprised of 26 (35.1%) cases and the second, designated as S2, of 48 (64.8%) cases. Table 3 displays the means (sds) of descriptive characteristics of S1 and S2. Subjects in the subtypes were similar on race/ethnicity, education, body mass index, cholesterol, and HbA1c. They did not differ on their use of psychotropic medications (71% in S1 and 62% in S2) but higher percents in S2 have current and lifetime depression (*p* < 0.05; 94% of members of S2 compared to 69% in S1).

**Table 3:** Comparison of selected characteristics of S1 and S2.

	S_1_ *n* = 26	S_2_ *n* = 48	^a^*p*-value of S_1_ vs. S_2_
Age (years, Mean (sd))	31.5 (6.36)	33.04 (7.82)	0.392
Ethnicity (%)			
Hispanic	9 (34.62%)	24 (50.00%)	0.488
Non-hispanic Asian	0 (0.00%)	1 (2.08%)	
Non-hispanic black	8 (30.77%)	12 (25.00%)	
Non-hispanic white	9 (34.62%)	10 (20.83%)	
Non-hispanic other	0 (0.00%)	1 (2.08%)	
Education (%)			
Less than 12th grade	0 (0.00%)	1 (2.08%)	0.5656
HS diploma or GED	11 (42.31%)	16 (33.33%)	
2 years college, AA degree	5 (19.23%)	17 (35.42%)	
4 years college, BA degree	9 (34.62%)	12 (25.00%)	
Masters degree	1 (3.85%)	2 (4.17%)	
Doctoral degree	0 (0.00%)	0 (0.00%)	
Body mass index (mean (sd))	29.59 (5.38)	30.43 (5.18)	0.515
Cholesterol (mean (sd))			
HDL Cholesterol	47.51 (11.80)	47.97 (11.72)	0.872
LDL Cholesterol	113.64 (38.28)	107.26 (28.43)	0.419
Psychiatric diagnoses			
% Current major depression	8 (30.77%)	33 (68.75%)	0.0017
% Current no alcohol use	11 (42.31%)	20 (41.67%)	0.9574
% Lifetime depression	18 (69.23%)	45 (93.75%)	0.0128
Psychotropic medication			
% on medication	17 (70.83%)	24 (61.54%)	0.4523

^a^Based on chi-square or ANOVA tests comparing S1 with S2 on the characteristic.

S1 and S2 differ in clinical severity. The mean CAPS score of individuals in S1 is 54.3 (sd 6.6), and of those in S2 is 75.6 (sd 14.6). Those in S2 compared to S1 have significantly higher mean severity scores on almost all individual items of the 16 clinical scales (see Fig. S2). In the principal component analysis of the total scale and subscale scores of the clinical scales used for subtyping, PC1 accounted for 64% of the total variance and had fairly equal factor weights on all of the clinical scale scores. Each of the additional principal components accounted for ≤5% of the total variance (see Table S5). All summary scale scores statistically differed between groups overall and in most pairwise comparisons of groups as noted in Table 2. In particular, the mean PC1 score of those in S2 was three times that of the S1 group.

### Validation

Figure S1b, a two-dimensional diffusion mapping of the RF distance scores based on a multidimensional scaling procedure, displays clear separation of HC from S1 and S2 and indicates greater dispersion among the members of S1 compared to S2. The silhouette scores reflect this: S1 = −0.25, S2 = 0.54 in comparison to HC = 0.76 (see Fig. S1a). The RF used to obtain the proximity of cases was scored with the external validation sample yielding an AUC of 0.99. The ability to classify persons into subtypes based on biological and other variables distinct from the subtyping variables with high accuracy reported below, a form of concurrent validity, was considered supportive of the external validity of the subtypes.

### Biomarker classification of PTSD subtypes

The subtypes were accurately classified with a multi-omic panel of 342 biological markers that RF analyses substantially reduced to 71 unique “important” biological markers over all intergroup comparisons. Table S4 displays the AUCs for each intergroup comparison for each feature class and the feature classes pooled. The AUCs and the number of important features in each of the down-selected intergroup models are: S1 vs. S2: AUC = 0.819, number of markers = 10; HCs vs. S1: AUC = 0.911, number of markers = 23; HCs vs. S2: AUC = 0.922, number of markers = 37. Supplementary Table S6 displays the set of important multi-omic features identified in any of the intergroup contrasts (*n* = 71), the mean (sd) values for each subtype, and HCs.

Table 4 displays for the top 5 markers in each comparison (*n* = 15 markers) the results of significance tests of equality of the means between the groups in an intergroup classification. Indicated is whether the marker found to be significant was up or down-regulated in the more severe group, as determined by a comparison of mean values. For comparisons of S2 vs. HC and of S1 vs. HC, mean values of the top five features of the groups significantly differed, and all markers except lactate were downregulated in the more severe group. The top five markers in the RF comparing S2 to HC were lactate, CG20720918 and CG03267026 and miR-106b.3p, and miR-93.3p. For S1 vs. S2 only one marker, cg13034868 statistically differed between groups indicating downregulation in the severe group.

**Table 4:** Significance* of difference in mean marker values and direction of dysregulation in the more severe group in intergroup comparison for top 5 RF important^a^ biomarkers.

RF	S_2_ vs. S_1_	S1 vs. HCs	S2 vs. HCs	PTSD cases vs. HCs
# markers retained in RF	**10**	**23**	**37**	**48**
hsa.miR.93.3p			**<0.001 (4) ▼**	**<0.001 (1) ▼**
cg01882498		**0.003 (1) ▼**		
cg01208318		**<0.001 (4) ▼**		**<0.001 (2) ▼**
cg20720918			**<0.001 (1) ▼**	<**0.001 (3) ▼**
cg13034868	**0.001 (1) ▼**			
hsa.miR.106b.3p			**<0.001 (2) ▼**	**<0.001 (4) ▼**
lactate			**<0.001 (3) ▲**	
cg23594345		**<0.001 (2) ▼**		**<0.001 (5) ▼**
cg15687973		**0.001 (3) ▼**		
cg18171204	0.594 (2)			
hsa.miR.127.3p		**<0.001 (5) ▼**		
cg03267026			**0.002 (5) ▲**	
cg06751007	0.211 (3)			
eosino	0.106 (4)			
APOF.SGV	0.023 (5)			

▼ = for significant markers, downregulated in more severe group based on comparison of mean values.

▲ = for significant markers, upregulated in more severe group based on comparison of mean values.

*FWER, Bonferroni corrected *p* < 0.0025, *p*-value bolded.

^a^() = importance rank in RF.

### Classification into subtypes by other feature classes

When all feature classes were included in RFs for intergroup classification models, the AUCs only slightly increased over that for biomarkers alone. For example, when all classes are combined (see Table S4), the AUC of S2 vs. HC is 0.968, only a 5% increase over that obtained with blood biomarkers alone (0.922). No feature class alone provided greater accuracy than did the biomarker class alone. In the classification model of S2 vs. HCs, neuro-cognitive functions and psychiatric history had AUCs of 0.72 and 0.84 with vocabulary test scores and lifetime major depression ranked as the most important features in these RF (see Table S7).

## Discussion

S2 is clinically a more severely ill group, with clinical item scores indicating greater severity for almost every item of all clinical scales considered, underscored by a mean total CAPs score difference between subtypes of 21 points (54.3 vs. 75.6). Almost all in S2 reported having lifetime major depression. Among the six feature classes studied, multi-omic biomarkers provided the highest accuracy for classification into subtypes, suggesting that clinically defined subtypes have strong biological underpinnings. Subtyping into clinical severity groups and high accuracy classification models using multi-omic blood markers provides strong support for biological associations with levels of PTSD severity.

That biological markers can be used for the classification of PTSD vs. HC was previously reported by our consortium in Dean et al.^8^ using 28 biomarkers. Using our RF methods and down selecting from the same 342 markers, in the present study we obtained an RF with 48 biomarkers, six of which are in common with the original 28, resulting in a high AUC of 0.91. That our current markers do not totally overlap with the original 28 may be explained in part by strong correlations among many molecular markers. To test this assumption we examined the canonical correlations of the 22 non-overlapping features from our earlier study with the 42 non-overlapping markers from our current study. The first three canonical correlations were 0.94, 0.90, and 0.84, demonstrating high correlations between the two sets of markers.

There are several published reports on the possible biological connections of markers to PTSD. We discuss the top five most important markers in the RF of the most extreme severity group contrast, S2 vs. HC^37–48^ viewing this contrast as most likely to identify signature pathways. Lactate, an important marker, is elevated, as was also found in earlier studies of the SBC^8,37^. It also has been found to be elevated in anxiety and panic disorder^38^. Cerebral lactate level is elevated in patients with schizophrenia^39^. Lactate is a marker for anaerobic metabolism, which is frequently elevated during physiological stress such as hypoxia, infection, and inflammation. As PTSD is a multi-systemic disorder, it is not surprising that there are stress responses at the cellular level, which may be a cause or the result of PTSD meriting further investigation.

The other four top markers in the RF classifying S2 vs. HC are CG20720918 (GORASP2), CG03267026 (BRSK2), miR-106b, and miR-93. These are molecular markers that are differentially expressed in various neurological and psychiatric illnesses. Both GORASP2 and BRSK2 genes are involved in the endoplasmic reticulum (ER) stress response which often results in apoptosis. GORASP2 encodes Golgi reassembly-stacking protein 2, also called GRASP55 (Golgi reassembly-stacking protein of 55 kDa), a membrane protein important in maintaining the stacking of Golgi cisternae. DNA hypomethylation of GORASP2 is associated with medial temporal epilepsy^40^. The transcriptional level of GORASP2 is also found to be altered in patients with Alzheimer’s disease (AD)^41^. Copy number variation of GORASP2 has also been found in autism spectrum disorder (ASD)^42^. On the other hand, BRSK2 encodes brain-specific kinase-2 involved in apoptosis as an ER stress response and also has a role in axon development. BRSK2 has been recently found to be a strong risk gene for ASD^43^. Autoantigen against BRSK2 was also found in paraneoplastic limbic encephalitis^44^. The relationship between the two microRNAs, miR-106b and miR-93, and PTSD is more enigmatic. Micro RNAs are short single-stranded non-coding RNAs that regulate gene expression by binding to complementary sequences in their target mRNAs′ 3′-untranslated region (UTR), and also to a lesser extent to the 5′-UTR and coding regions. Both of these miRNAs have lower levels in the S2 subtype compared to the HC subjects. They are derived from the MCM7 (mini-chromosome maintenance) gene, which encodes a protein essential for the initiation of eukaryotic genome replication and belongs to the highly conserved miR-106b-25 cluster. The serum levels of miR-106b have been shown to be upregulated in both schizophrenia and bipolar disorder^45,46^, which is the opposite of our finding in the S2 subtype patients. On the other hand, serum miR-93 levels are significantly decreased in patients with AD^47^. It is possible that PTSD patients have significant ER stress at the subcellular level, although the exact mechanism merits further investigation. On the other hand, as microRNAs target multiple molecular pathways simultaneously based on their sequence homology, not on function, it is not surprising that similar changes in serum microRNA levels may be found in different diseases. The mechanism of action of microRNAs is complex and involves a variety of signaling pathways and target genes. For example, studies have shown that miR-106b and miR-93 induce the migration, invasion, and proliferation of cancer cells and simultaneously enhance the activity of the phosphatidylinositol-3 kinase (PI3K)/AKT pathway^48^. Whether the PI3K/AKT pathway is disturbed in PTSD, other neurological or psychiatric diseases and cancers remains unclear. However, it is likely that PTSD involves dysregulation of multiple pathways.

## Limitations

Our discovery sample was modest in size, limiting the identification of subtypes to two groups. Its restriction to males limits its generalizability. Our findings require replication in larger and more diverse samples, including female veterans and trauma-exposed civilians with and without PTSD. Our participants were 6–10 years post their index traumatic events, not allowing for subtyping closer in time to exposure. In addition, we did not include participants with other psychiatric disorders, leaving unanswered the question of whether our findings generalize to severity subgroups of other disorders. While considering a large set of multi-omic blood markers biomarkers, we did not include structural, functional, molecular, and EEG neuroimaging markers, and to do so might have increased classification accuracy of the PTSD subtypes modeled. Identified biomarkers differed between models, but might be highly correlated or otherwise multivariately related. Further exploration is required of identified multivariate marker profiles to clarify their relationship to each other and to severity.

## Conclusions

Our findings suggest that male veterans with military service-related PTSD with more severe symptoms across a wide range of clinical scales assessing PTSD and its comorbidities can be biologically distinguished from HC on blood biomarkers most of which are mRNA and methylation markers. Down-regulation of these markers in relationship to severity is suggested while an increase in mean serum lactate levels was noted. There is evidence in the literature that the top five biological markers that differentiated the more severe group from HC are also associated with various neurological and psychiatric illnesses. Biological markers outperform other potential predictor classes—current medical comorbidities, neurocognitive functioning, demographics, pre-military trauma, and psychiatric history—as evidenced by their substantially higher AUCs.

The importance of the associations among the biological features and clinical severity is likely a reflection of a complex disorder. Studies of the mechanistic pathways and similarities with other illnesses may provide directions for future research for understanding the biological basis of PTSD. The use of well-calibrated biological markers for PTSD subtype classification can help bring symptom-based diagnosis to a more objective laboratory basis and facilitate the development of treatments targeted to severity.

## Disclaimer

The opinions or assertions contained herein are the private views of the authors and are not to be construed as official, or as reflecting true views of the Department of the Army or the Department of Defense.

## Supplementary information

Supplemental Material
