# Supplementary material for: Utilization of machine learning for identifying symptom severity military-related PTSD subtypes and their biological correlates
Source: Transl Psychiatry. 2021 Apr 20;11:227. doi: 10.1038/s41398-021-01324-8 (PMC8058082; doi:10.1038/s41398-021-01324-8)
Supplement: Supplementary file 1 — Supplemental Material [file 41398_2021_1324_MOESM1_ESM.docx]

**SUPPLEMENT: tables and figures**

**Table S.1 : Clinical Scale Abbreviations**

**Table S.2: 342 candidate biology biomarkers**

**Table S.3: Number of candidate biomarkers by biological type**

**Table S.4: AUCs for each feature class and all six classes combined for each intergroup comparison**

**Table S.5: PCA Analysis: Component loadings on PC1 and PC2 of subtyping total and subscale scores**

**Table S.6 : Subgroup Means (sds) of important biology biomarkers (n=71) identified in intergroup RFs**

**Table S.7: Mean (sds) values of variables entering RF in feature classes of**

**neuro-cognition and past psychiatric diagnoses**

**Figure S.1: 1a. Silhouette Plot ; 1b. Diffusion Plot**

**Figure S.2: Plots of comparison of individual item**

**means for each subtyping scale for S1, S2 and HCs**

**Table S.1 : Clinical Scale Abbreviations**

| Scales | Subscales | Description |
| --- | --- | --- |
| PC1 |  | Principal Component 1 |
| CAPS |  | Clinician Administered PTSD Scale |
| PCL |  | PTSD Symptom Check List, DSM-IV |
| SCL-90 |  | Symptom Checklist-90 |
|  | SCLSOM | Somatization |
|  | SCLOC | Obsession/ Compulsion |
|  | SCLINT | Interpersonal sensitivity |
|  | SCLDEP | Depression |
|  | SCLANX | Anxiety |
|  | SCLHOS | Hostility |
|  | SCLPHOB | Phobias |
|  | SCLPAR | Paranoid ideation |
|  | SCLPSY | Psychoticism |
|  | SCLGSI | Global severity index |
|  | SCLPST | Positive symptoms |
|  | SCLPSDI | Positive symptom distress index |
| PSQI |  | Pittsburgh Sleep Quality Inventory |
| BDI |  | Beck Depression Inventory |
| PANAS PA |  | Positive Affect |
| PANAS NA |  | Negative Affect |
| MCS |  | Missouri Combat Scale |
| STAX |  | Spielberger State –Trait Anger Expression Inventory |
| PSS |  | Perceived Stress Scale |
| PSQI |  | Pittsburgh Sleep Quality Inventory |
| BDI |  | Beck Depression Inventory |
| PDEQRV |  | Dissociative Experiences rater |
| PDEQEV |  | Dissociative Experiences self |
| PDI rv |  | Peritraumatic Distress Inventory rater |
| PDI ev |  | Peritraumatic Distress Inventory self |
| ASI |  | Anxiety sensitivity index |
| ERS |  | Emotion regulation scale |
| SF12 |  | Short Form Health Survey (SF-12) |
|  | PF T | Physical Functioning |
|  | RP T | Role Limitation Physical |
|  | BP T | Pain |
|  | GH T | General Health |
|  | VT T | Vitality |
|  | RE T | Role Limitation Emotional |
|  | SF T | Social Functioning |
|  | MH T | Mental Health |

**Table S.2: 342 candidate biology biomarkers**

| cg00022594 | hsa.let.7a.1.5p | X.15anhydroglucitol15AG |
| --- | --- | --- |
| cg00436969 | hsa.let.7b.5p | X.2hydroxypalmitate |
| cg00567043 | hsa.let.7c.5p | X.3hydroxydecanoate |
| cg00739770 | hsa.let.7d.3p | X.5oxoproline |
| cg00780364 | hsa.let.7e.5p | X.7alphahydroxy3oxo4cholestenoate7Hoca |
| cg00792184 | hsa.let.7f.2.5p | X107503236 |
| cg00800038 | hsa.let.7g.5p | X107503313 |
| cg01160882 | hsa.let.7i.5p | X30683756 |
| cg01208318 | hsa.miR.100.5p | X30683880 |
| cg01358966 | hsa.miR.101.2.3p | X3323909 |
| cg01882498 | hsa.miR.103a.1.3p | X44864458 |
| cg02196619 | hsa.miR.103a.2.3p | X45411864 |
| cg02643835 | hsa.miR.106a.5p | X47209677 |
| cg02886208 | hsa.miR.106b.3p | X49372127 |
| cg03045169 | hsa.miR.106b.5p | X49372131 |
| cg03065308 | hsa.miR.107.3p | X49372157 |
| cg03267026 | hsa.miR.126.3p | X49372162 |
| cg03292149 | hsa.miR.127.3p | X63422568 |
| cg03405026 | hsa.miR.1296.5p | X6707521 |
| cg03433241 | hsa.miR.1301.3p | X6710721 |
| cg04112106 | hsa.miR.1307.3p | X70672835 |
| cg04768958 | hsa.miR.133a.1.3p | X70672874 |
| cg05254646 | hsa.miR.133a.2.3p | X70672983 |
| cg05452391 | hsa.miR.134.5p | X75938223 |
| cg05575921 | hsa.miR.143.3p | X75938289 |
| cg05644921 | hsa.miR.144.3p | X75938326 |
| cg05718652 | hsa.miR.148a.3p | X75938338 |
| cg06512815 | hsa.miR.152.3p | ch.9.137242878F |
| cg06582789 | hsa.miR.155.5p | cholest |
| cg06751007 | hsa.miR.17.5p | Cl |
| cg07004744 | hsa.miR.181c.5p | CLU.LFD |
| cg07387335 | hsa.miR.182.5p | CO2 |
| cg07462954 | hsa.miR.185.3p | Complement.C3 |
| cg07633119 | hsa.miR.186.5p | Complement.Factor.H |
| cg08110688 | hsa.miR.18a.5p | cortisol |
| cg08536977 | hsa.miR.18b.5p | CPN1.IVQ |
| cg08815261 | hsa.miR.191.5p | CPN2.LLN |
| cg08957001 | hsa.miR.192.5p | creatine |
| cg09629631 | hsa.miR.199a.1.5p | CRP.ESD |
| cg10104451 | hsa.miR.199a.2.5p | CTSS.GID |
| cg10424969 | hsa.miR.199b.5p | dihomolinoleate202n6 |
| cg10677959 | hsa.miR.20a.5p | dihomolinolenate203n3orn6 |
| cg10916401 | hsa.miR.22.3p | docosadienoate222n6 |
| cg11155865 | hsa.miR.223.5p | docosahexaenoateDHA226n3 |
| cg11896104 | hsa.miR.26b.5p | eicosapentaenoateEPA205n3 |
| cg11912591 | hsa.miR.28.3p | eicosenoate201 |
| cg11974820 | hsa.miR.301a.3p | elisa.cor1 |
| cg11993873 | hsa.miR.30a.3p | elisa.cordif |
| cg12243133 | hsa.miR.326.3p | elisa.corsup |
| cg12352896 | hsa.miR.328.3p | elisa.npy |
| cg13034868 | hsa.miR.329.2.3p | eosino |
| cg13074055 | hsa.miR.330.3p | F10.NCE |
| cg13176198 | hsa.miR.331.3p | GABR* * removed |
| cg13334396 | hsa.miR.338.3p | gammaglutamylisoleucine |
| cg13599649 | hsa.miR.3615.3p | gammaglutamyltyrosine |
| cg13720744 | hsa.miR.363.3p | GC.ELS |
| cg13736263 | hsa.miR.370.3p | ggt |
| cg13887966 | hsa.miR.376c.3p | glucose |
| cg14042143 | hsa.miR.409.3p | glutamine |
| cg14080518 | hsa.miR.423.3p | gsh |
| cg14178348 | hsa.miR.423.5p | gsh_gssg |
| cg14282850 | hsa.miR.424.3p | gssg |
| cg14569776 | hsa.miR.425.5p | HGFAC.VAN |
| cg14583127 | hsa.miR.4286.5p | HPX.SGA |
| cg14596589 | hsa.miR.4317.5p | A1BG.CEG |
| cg14633466 | hsa.miR.433.3p | ABM10 |
| cg14780132 | hsa.miR.4454.5p | ABM2 |
| cg15296538 | hsa.miR.4485.3p | ACTC1.DSY |
| cg15510325 | hsa.miR.484.5p | ACTC1.IIA |
| cg15553397 | hsa.miR.494.3p | acth1 |
| cg15687973 | hsa.miR.505.3p | acthdif |
| cg16163535 | hsa.miR.548au.5p | acthsup |
| cg16173229 | hsa.miR.598.3p | AFM.LPN |
| cg16335858 | hsa.miR.660.5p | alkphos |
| cg16956133 | hsa.miR.744.5p | alphahydroxyisovalerate |
| cg17078116 | hsa.miR.769.5p | alt |
| cg17128892 | hsa.miR.874.3p | AMBP.GEC |
| cg17137457 | hsa.miR.9.1.5p | APCS.IVL |
| cg17176946 | hsa.miR.93.3p | APOC2.TYL |
| cg18043888 | hsa.miR.93.5p | APOC4.AWF |
| cg18171204 | hsa.miR.99a.5p | APOC4.ELL |
| cg18184748 | hscrp | APOE |
| cg18187244 | hypoxanthine | APOF.SGV |
| cg18264219 | ic50 | arginine |
| cg19251850 | IL1RAP.VAF | B4GALNT2 |
| cg19257562 | iminodiacetateIDA | baso |
| cg19261518 | insulin | bdnf |
| cg19528338 | ITIH2.VQF | bdnf.serotonin |
| cg19675935 | ITIH4.LGV | C10orf107 |
| cg19976404 | lactate | C21orf125 |
| cg20004910 | lactate.citrate | C2orf3 |
| cg20578780 | lymph | C3 |
| cg20720918 | mch | C3.A2M |
| cg20850275 | mcv | C4BPB.LIQ |
| cg20989443 | MDC1 | C5.TDA |
| cg21011425 | monos | C9.LSP |
| cg21516384 | mpv | Ca |
| cg21668832 | neut | CA1.LYP |
| cg21718086 | OLFM1.LTG | CA1.VLD |
| cg22573359 | OR3A2 | carnitine |
| cg22661330 | ornithine |  |
| cg23094318 | pentadecanoate150 |  |
| cg23131950 | PGLYRP2.EFT |  |
| cg23233802 | PGLYRP2.GCP |  |
| cg23253752 | PLG.EAQ |  |
| cg23594345 | plt |  |
| cg23641264 | PPBP.GTH |  |
| cg24505167 | PRG4.DQY |  |
| cg24516362 | PRG4.GLP |  |
| cg24620508 | PTGDS.AQG |  |
| cg25414209 | pyruvate |  |
| cg25448062 | SERPINA7.GWV |  |
| cg25504868 | SHANK2 |  |
| cg25625104 | ST6GAL2 |  |
| cg25748226 | transurocanate |  |
| cg26088629 | triglyc |  |
| cg26454601 | tyrosine |  |
| cg26478582 | VASP.VQI |  |
| cg26505822 | vitc |  |
| cg26860703 | VTN.VDT |  |
| cg27030655 | WNT1 |  |
| cg27479267 |  |  |

**Table S.3: Number of candidate biomarkers by biological type**

| **Type of candidate biomarkers** | **Number** |
| --- | --- |
| Biometrics | 2 |
| Clinical Lab | 20 |
| Metabolomics | 27 |
| Methylation – Illumina array | 123 |
| Methylation – targeted sequencing | 33 |
| miRNA – plasma sequencing | 81 |
| Endocrine | 8 |
| Peptide – SRM | 38 |
| Protein – multiplex | 2 |
| Protein – bdnf | 1 |
| Small Molecule – Oxidation Byproducts | 4 |
| Nonlinear feature combinations | 3 |
| **Total** | **342** |

**Figure S.1:**

**Silhouette Plot 1a** A measure of how proximate an object is to other members of its cluster (cohesion) compared to members in other clusters (separation). The score ranges from −1 to +1, **
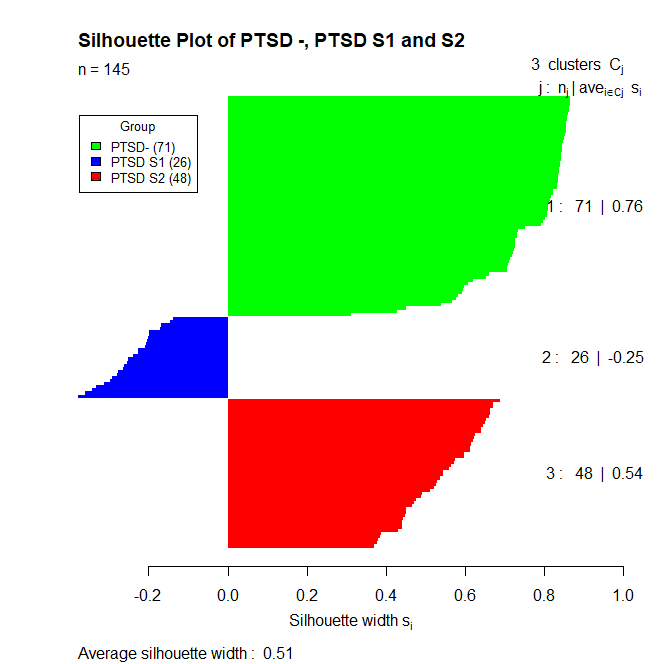
**

**1.b Subtype Diffusion Map Plot**


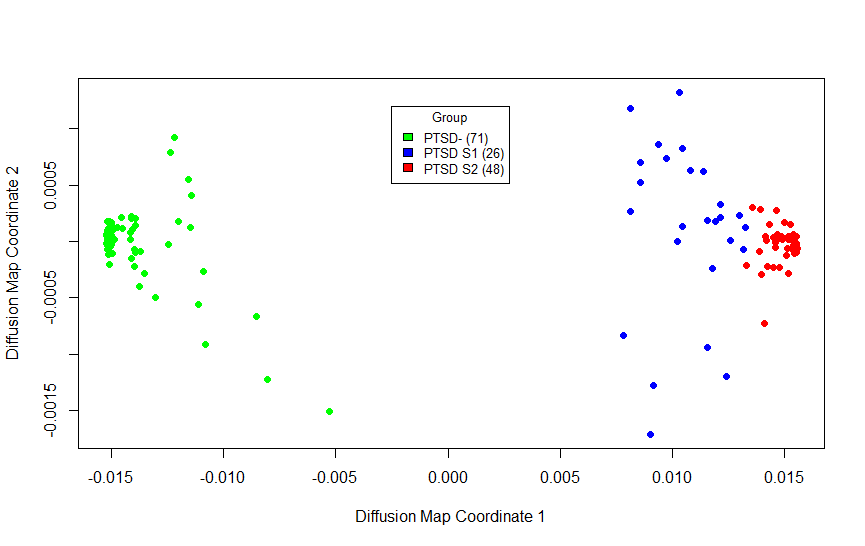


**Figure S.2: Plots of Comparison of individual item**

**means for each subtyping scale for S1, S2 and HCs**

**
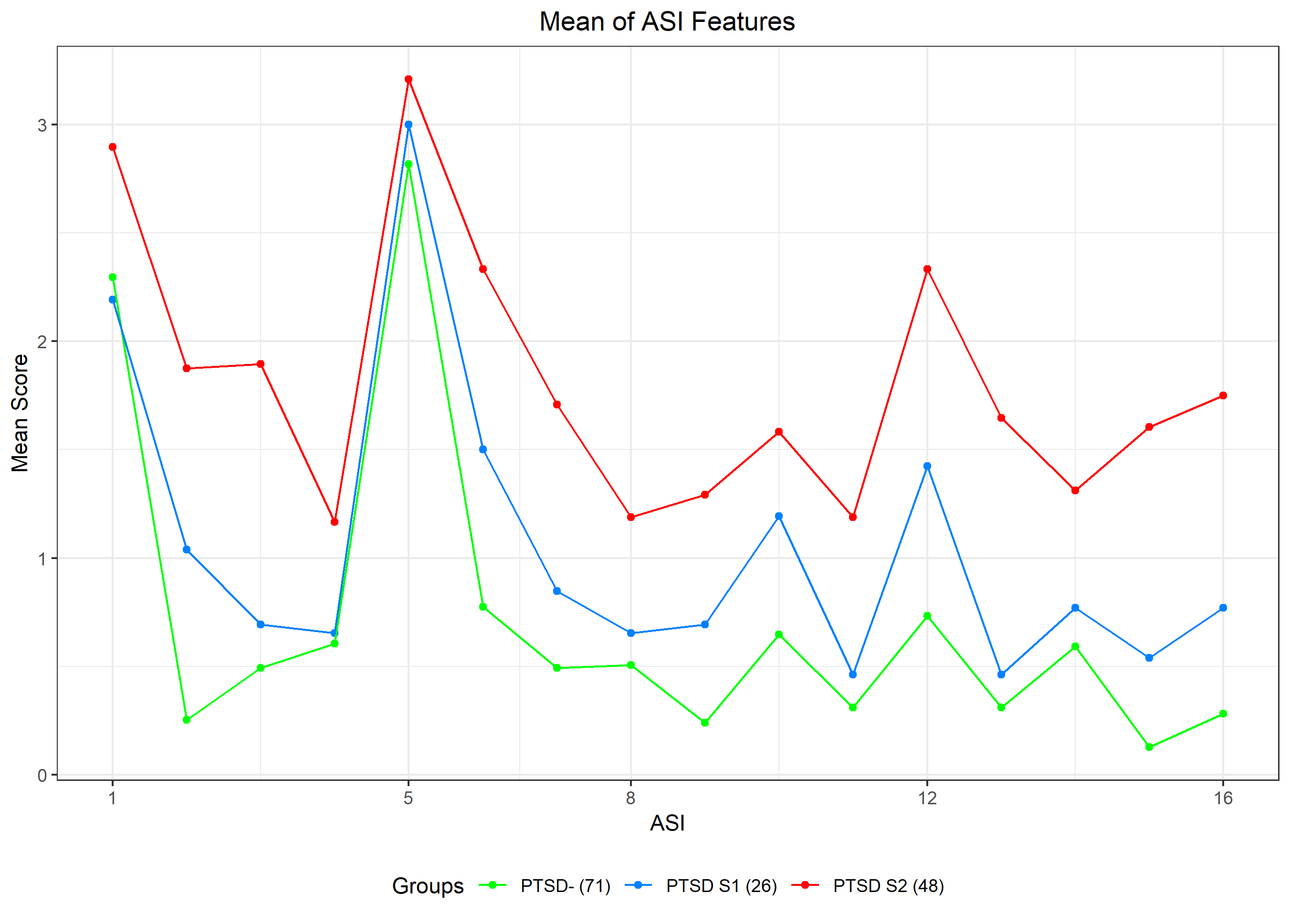

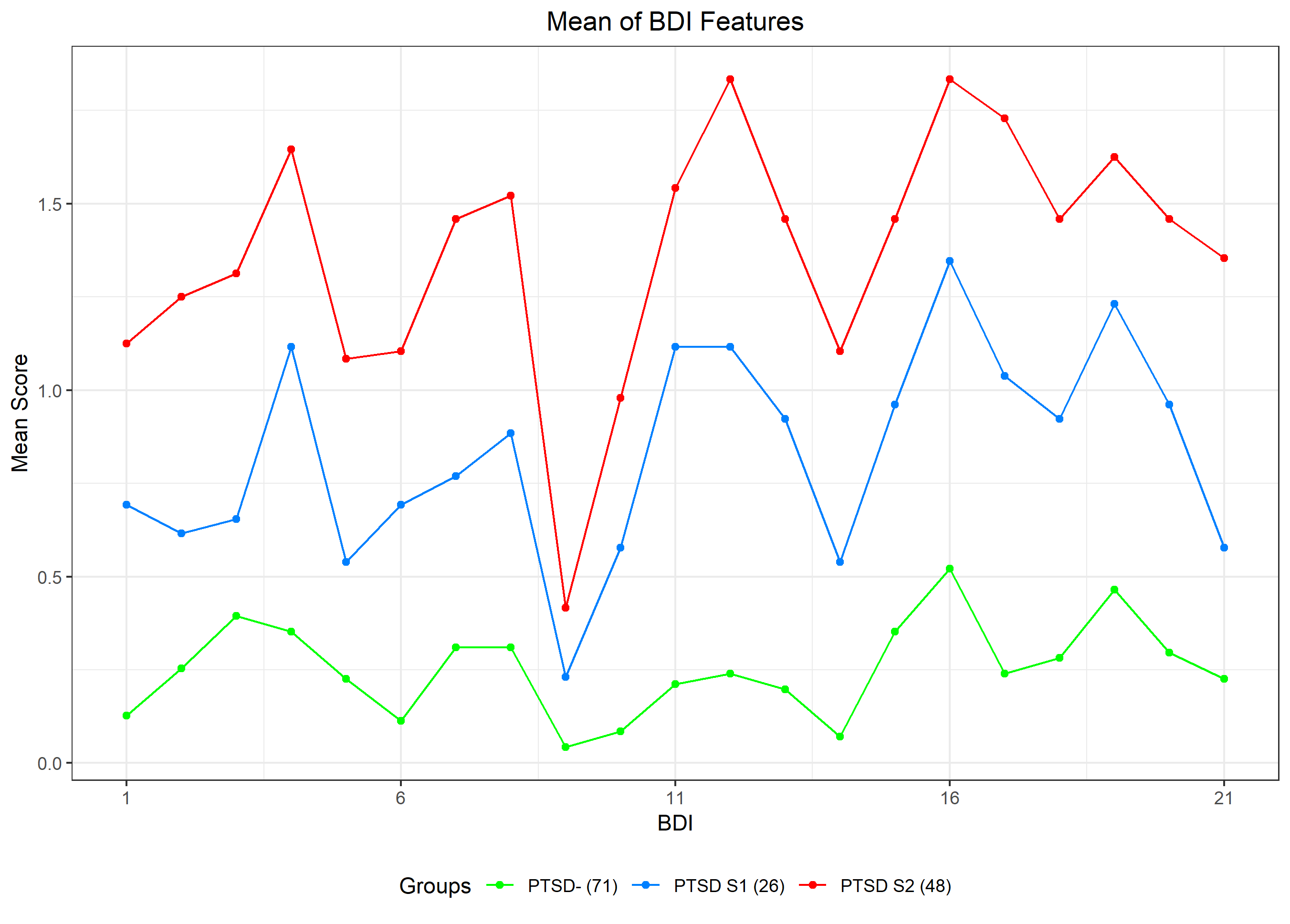

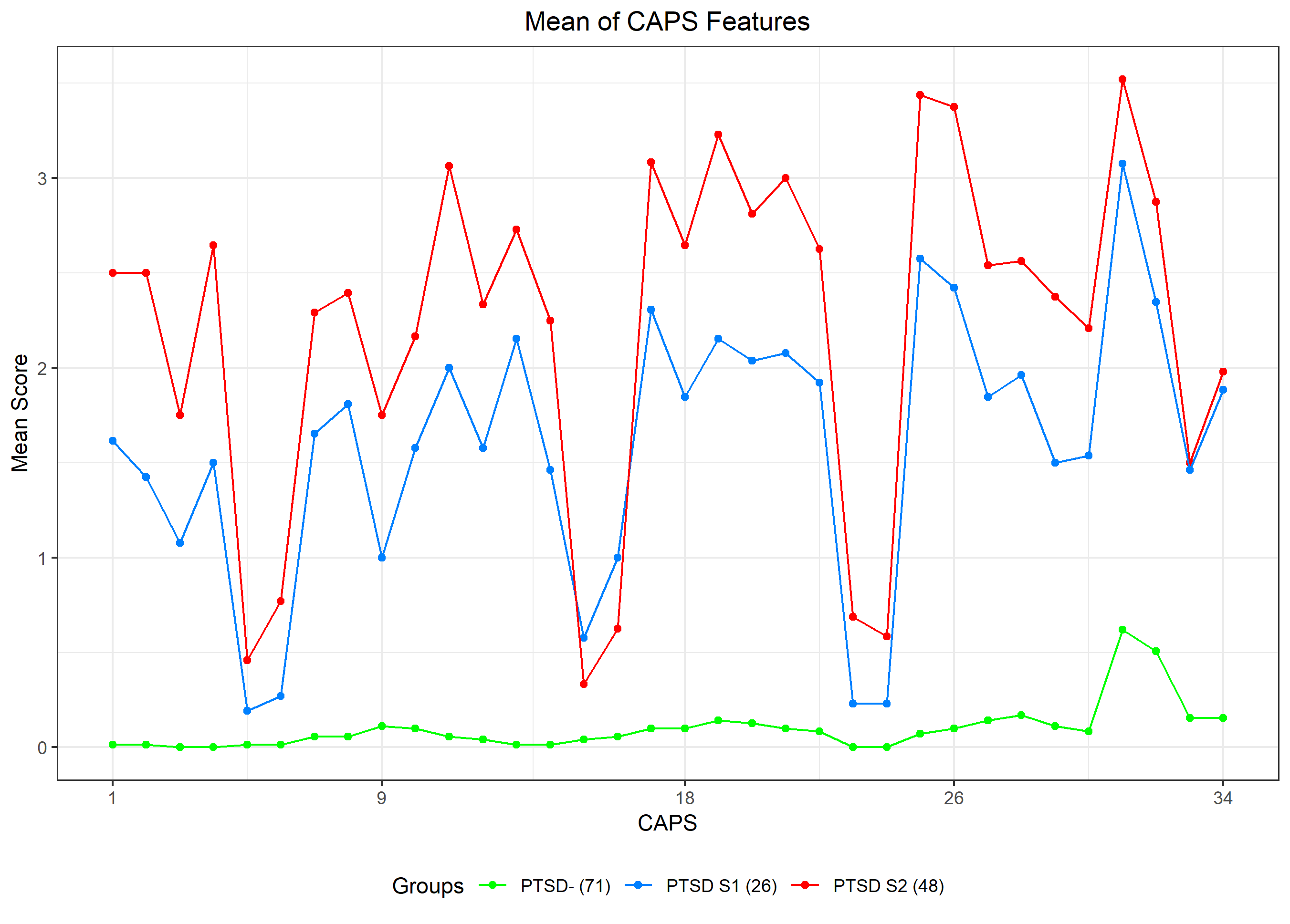
**

**
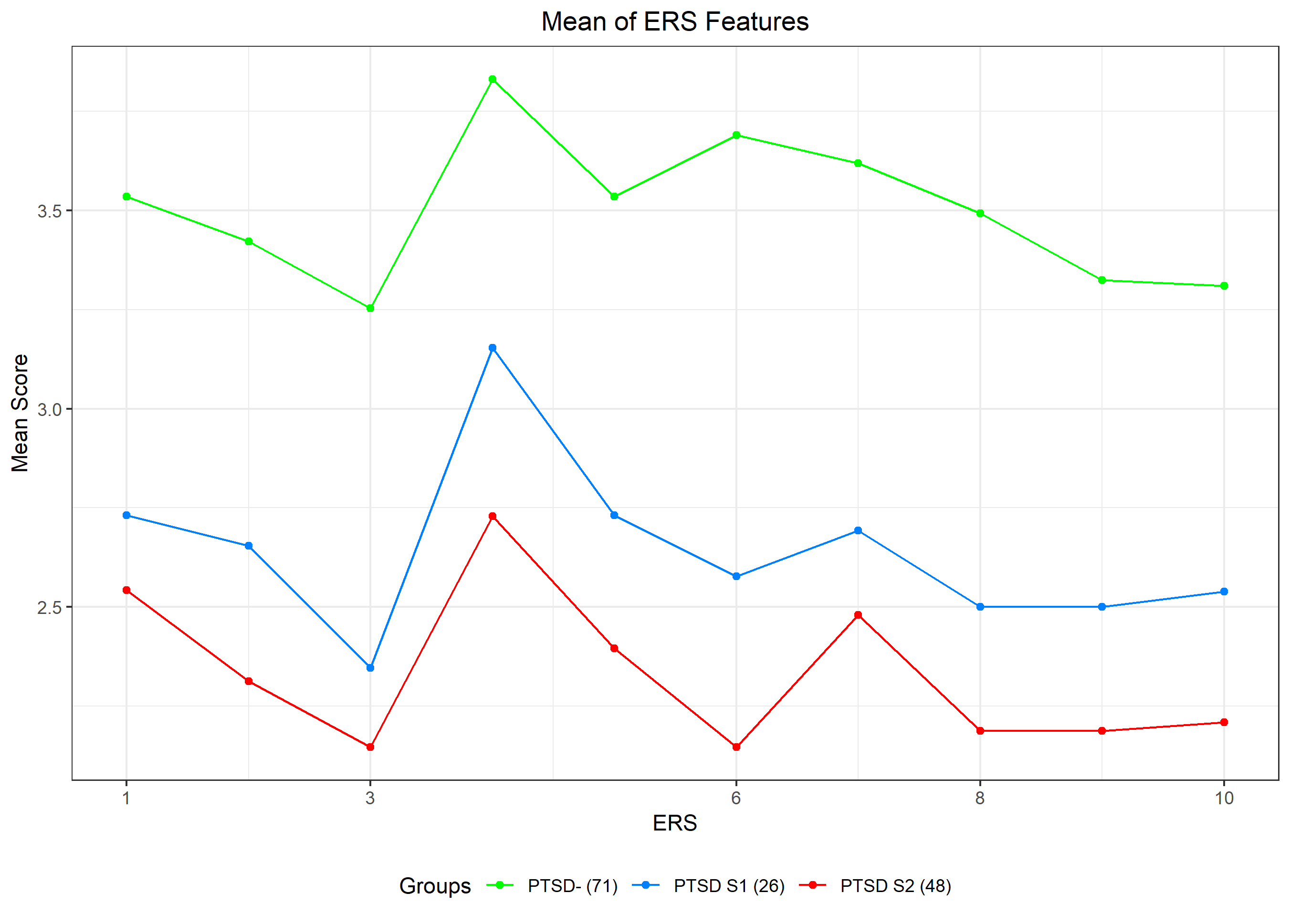
**

**
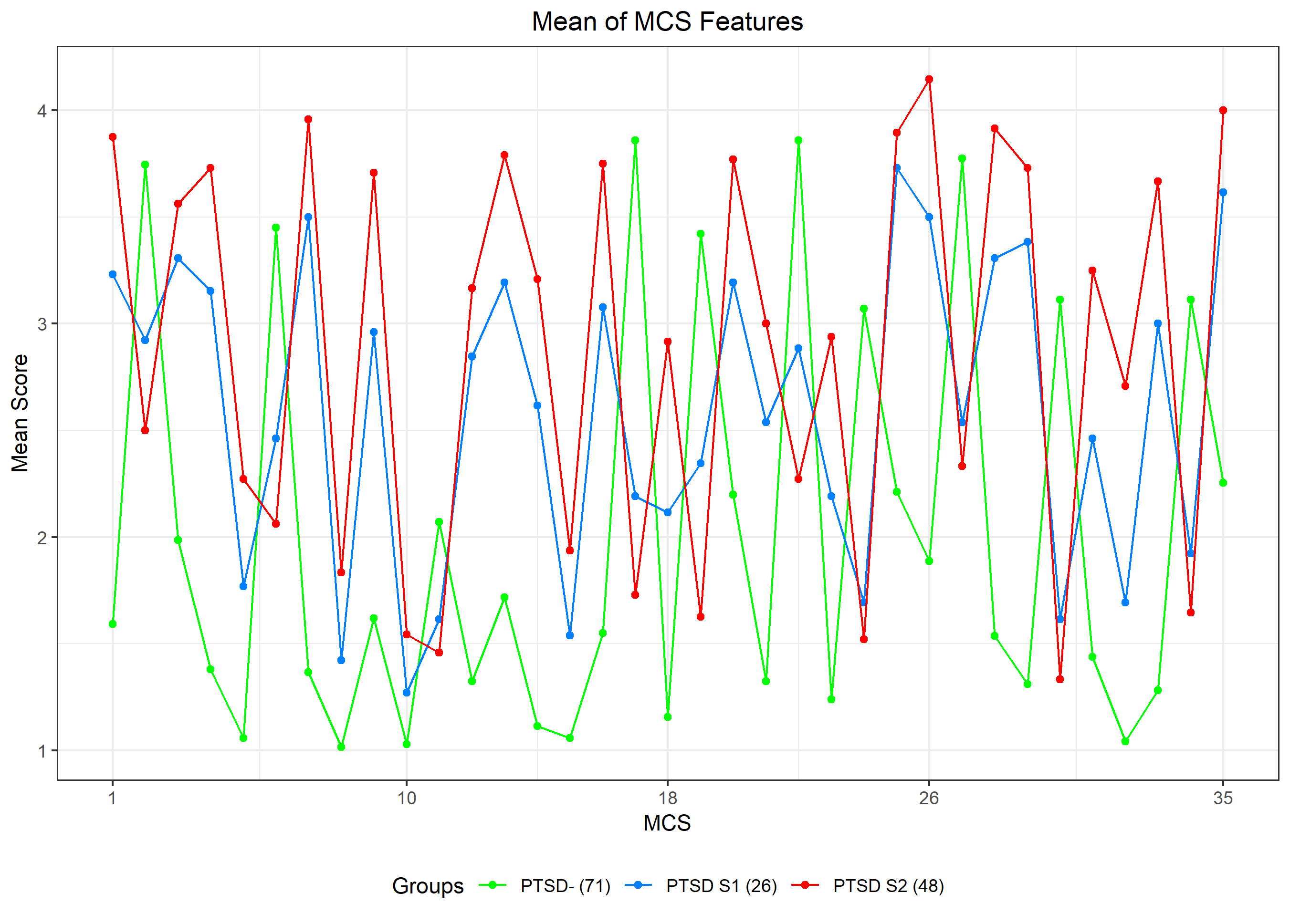

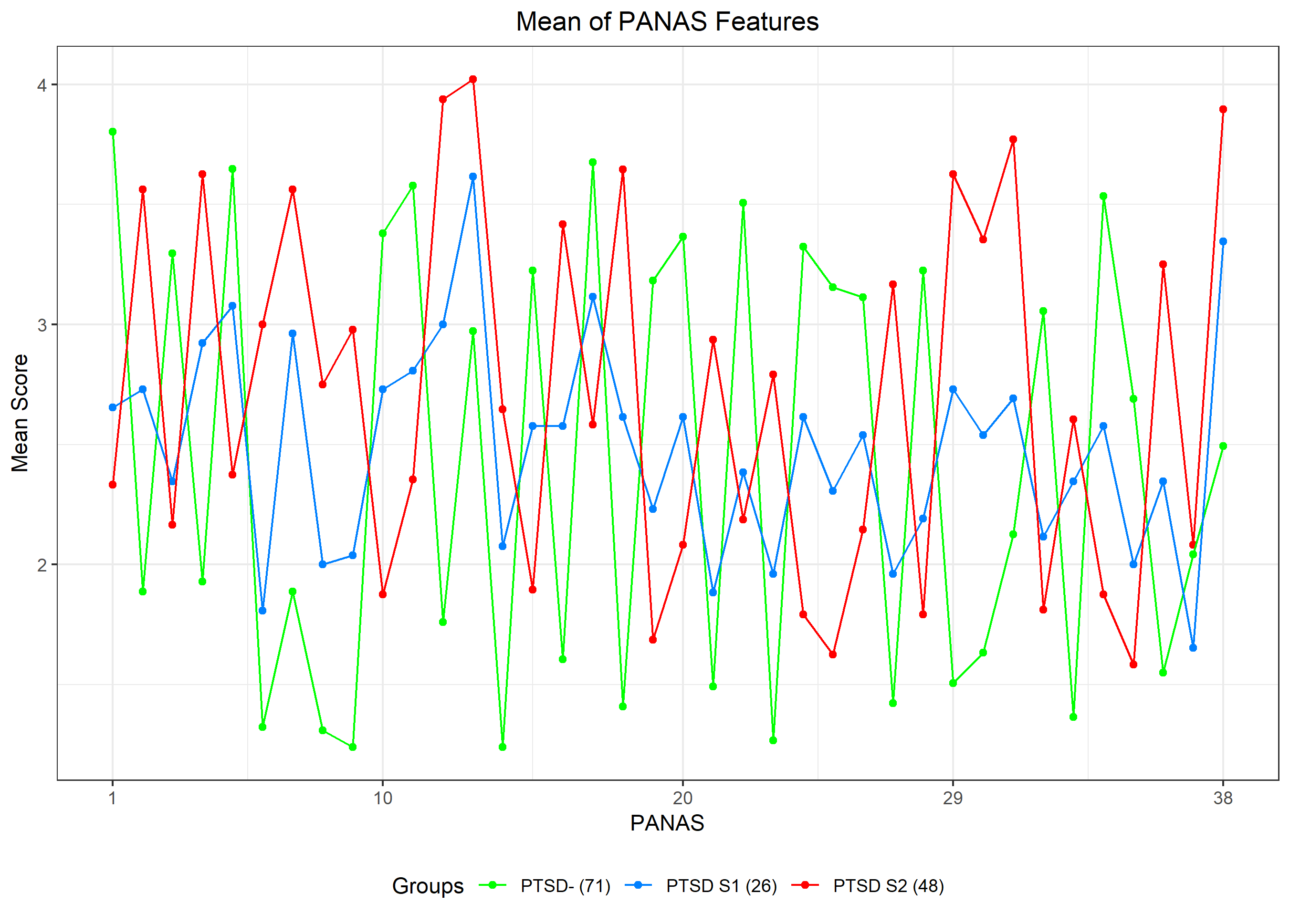
**

**
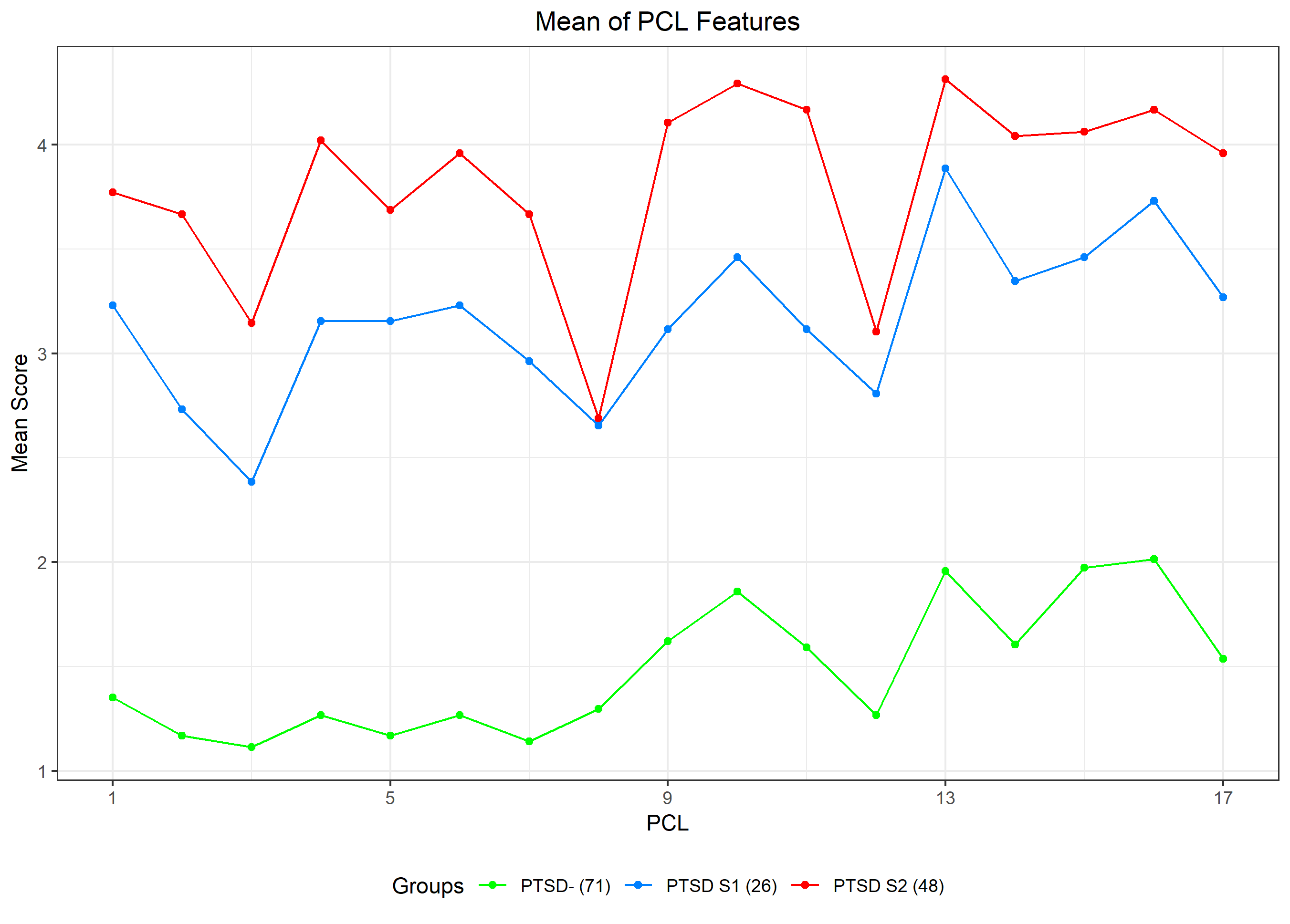
**

**
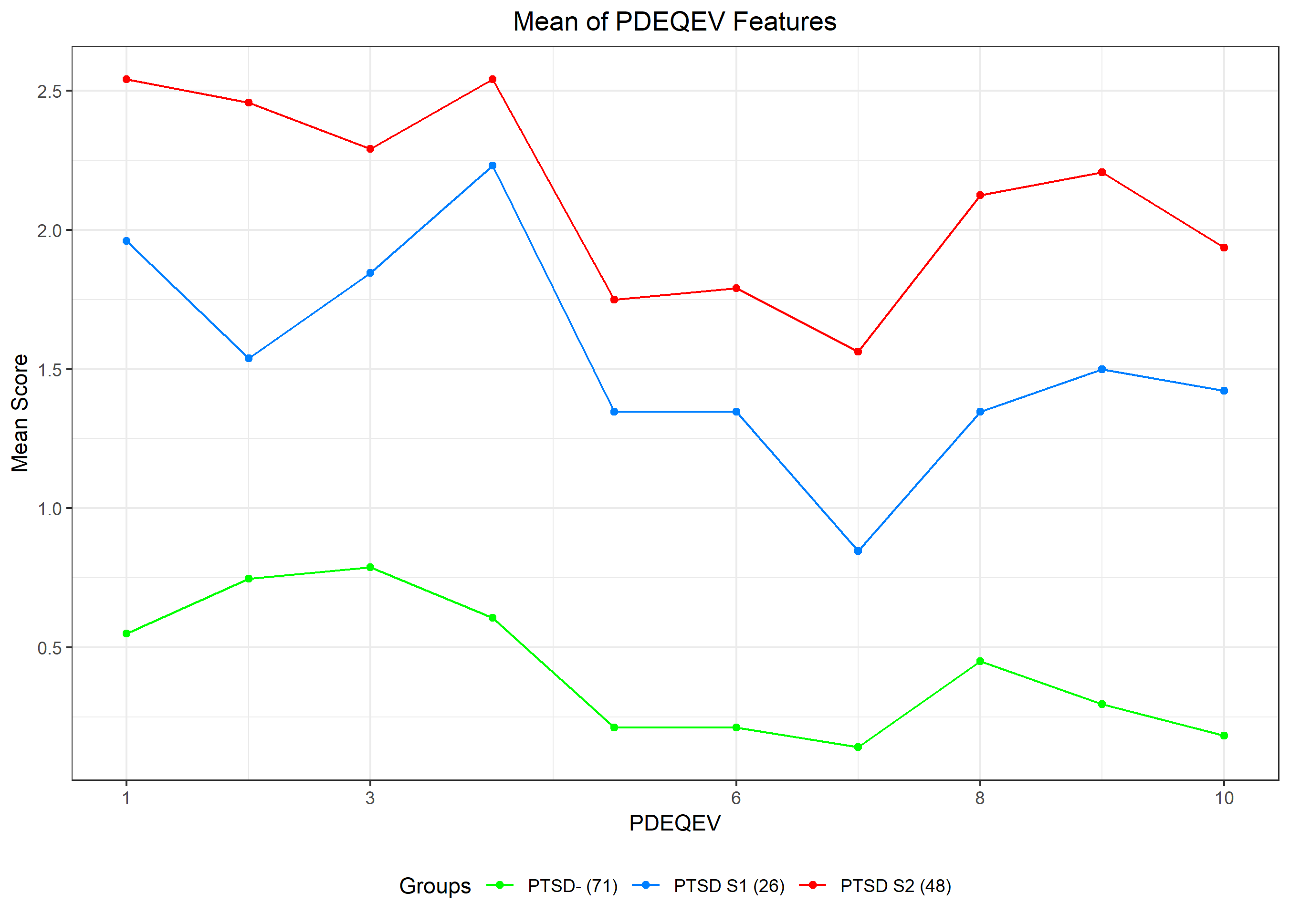
**

**
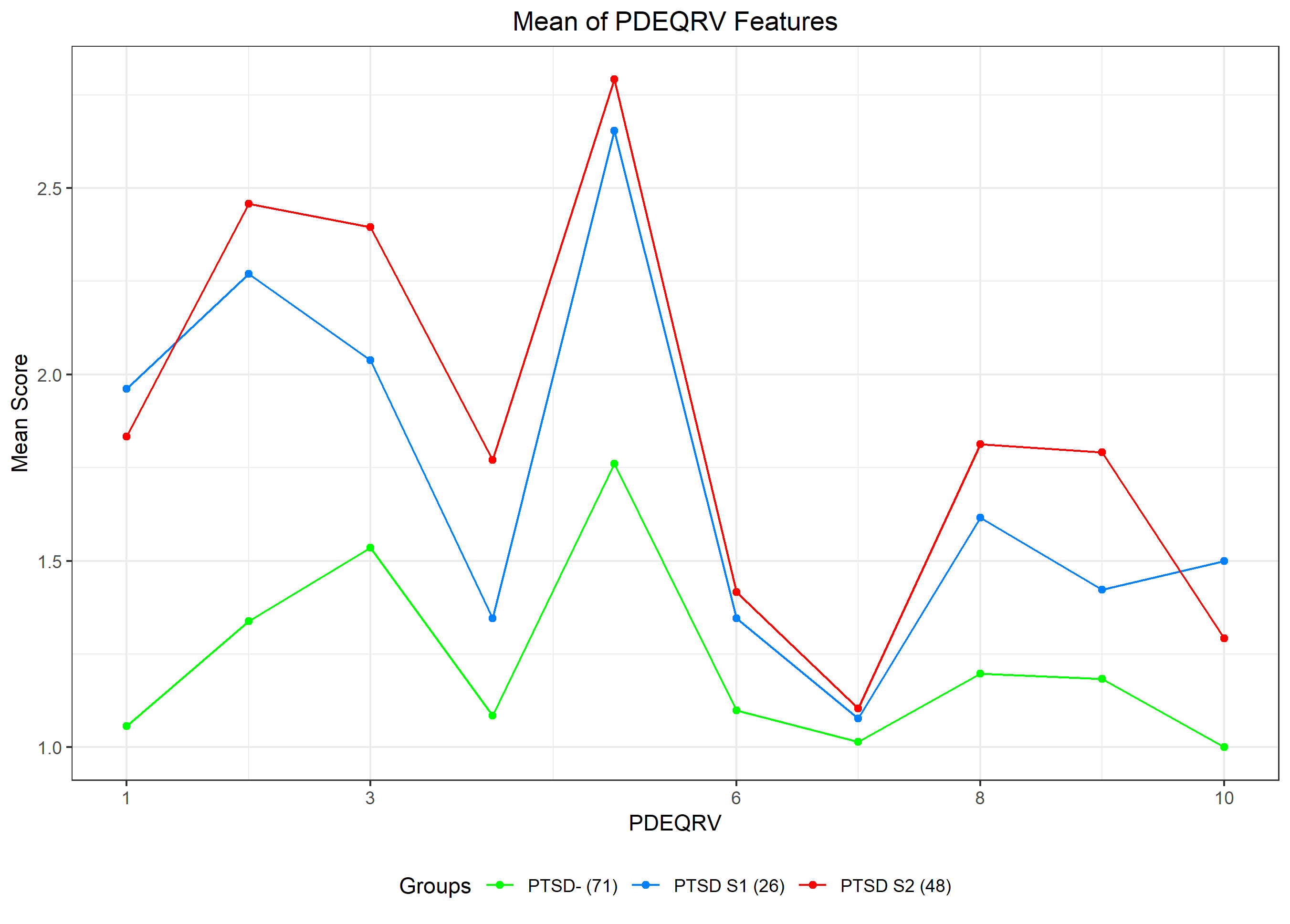
**

**
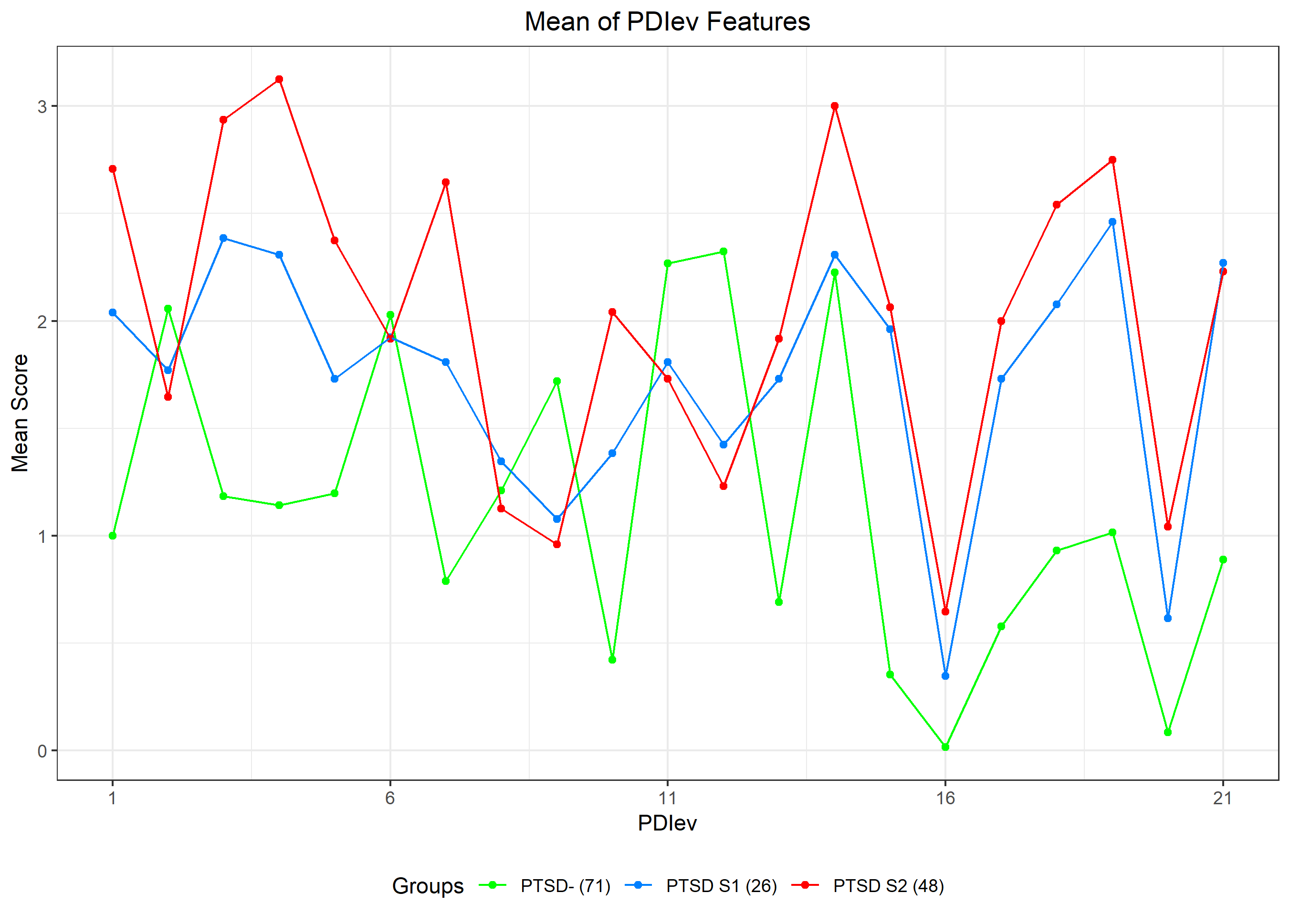
**

**
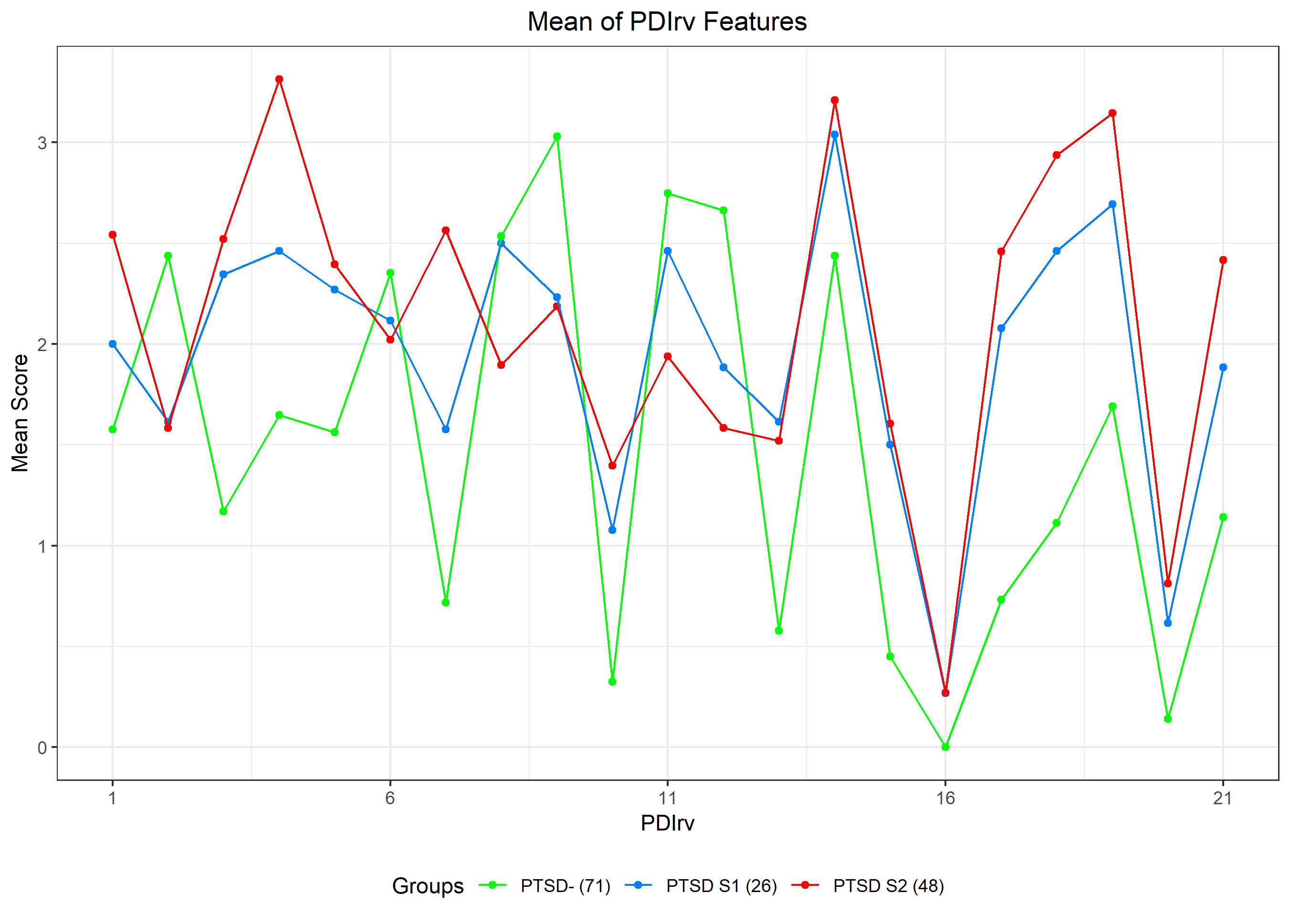
**

**
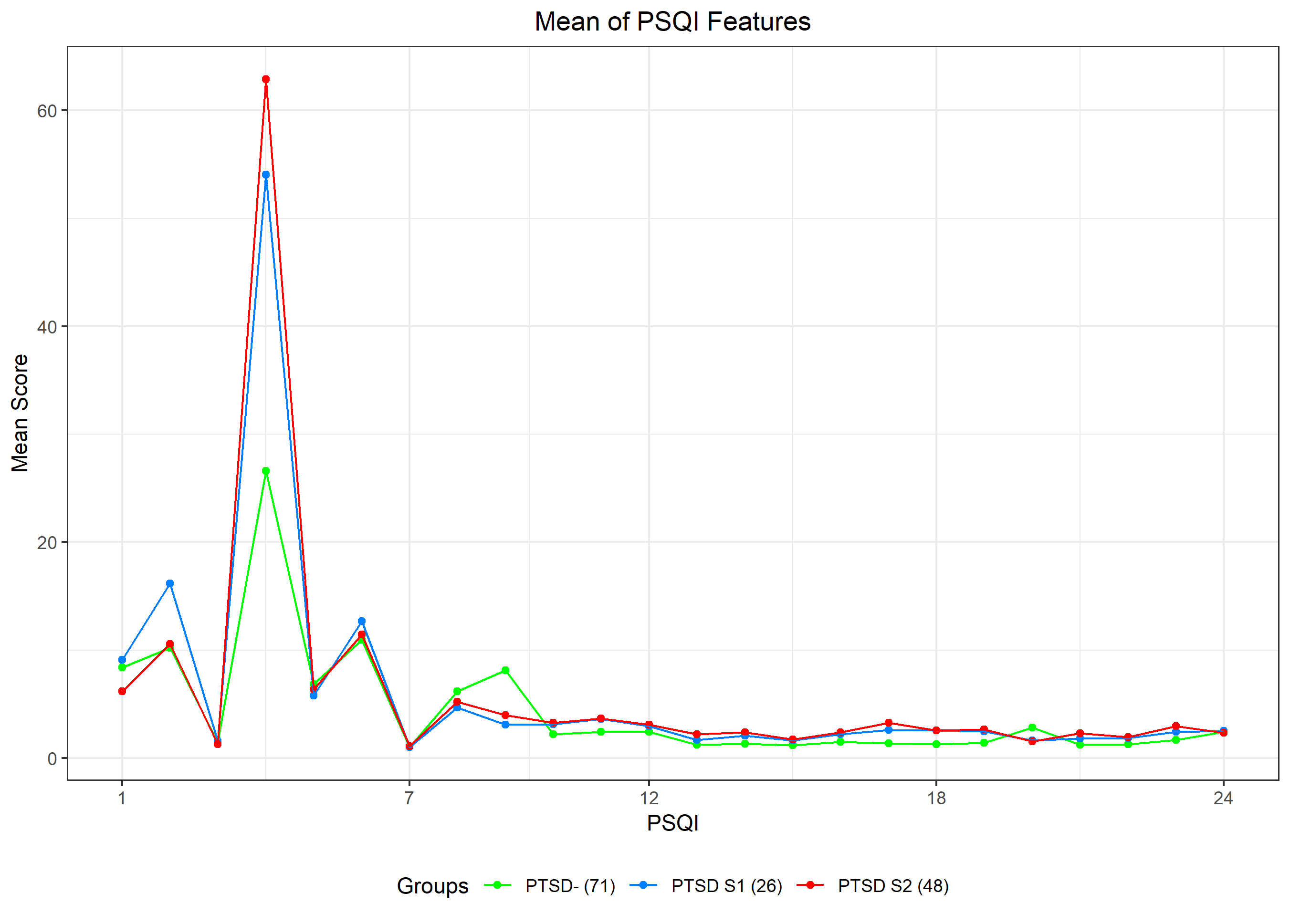
**

**
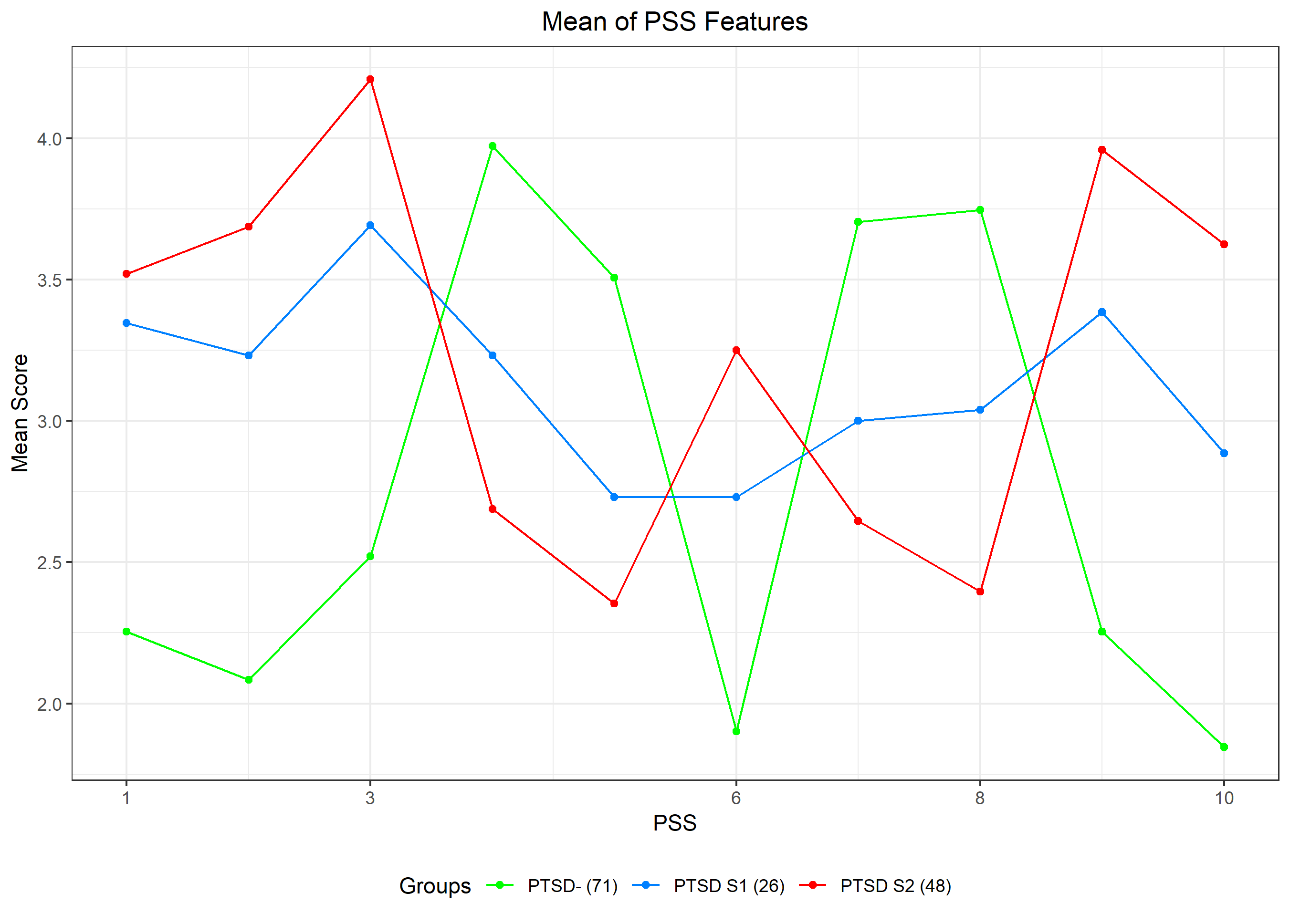

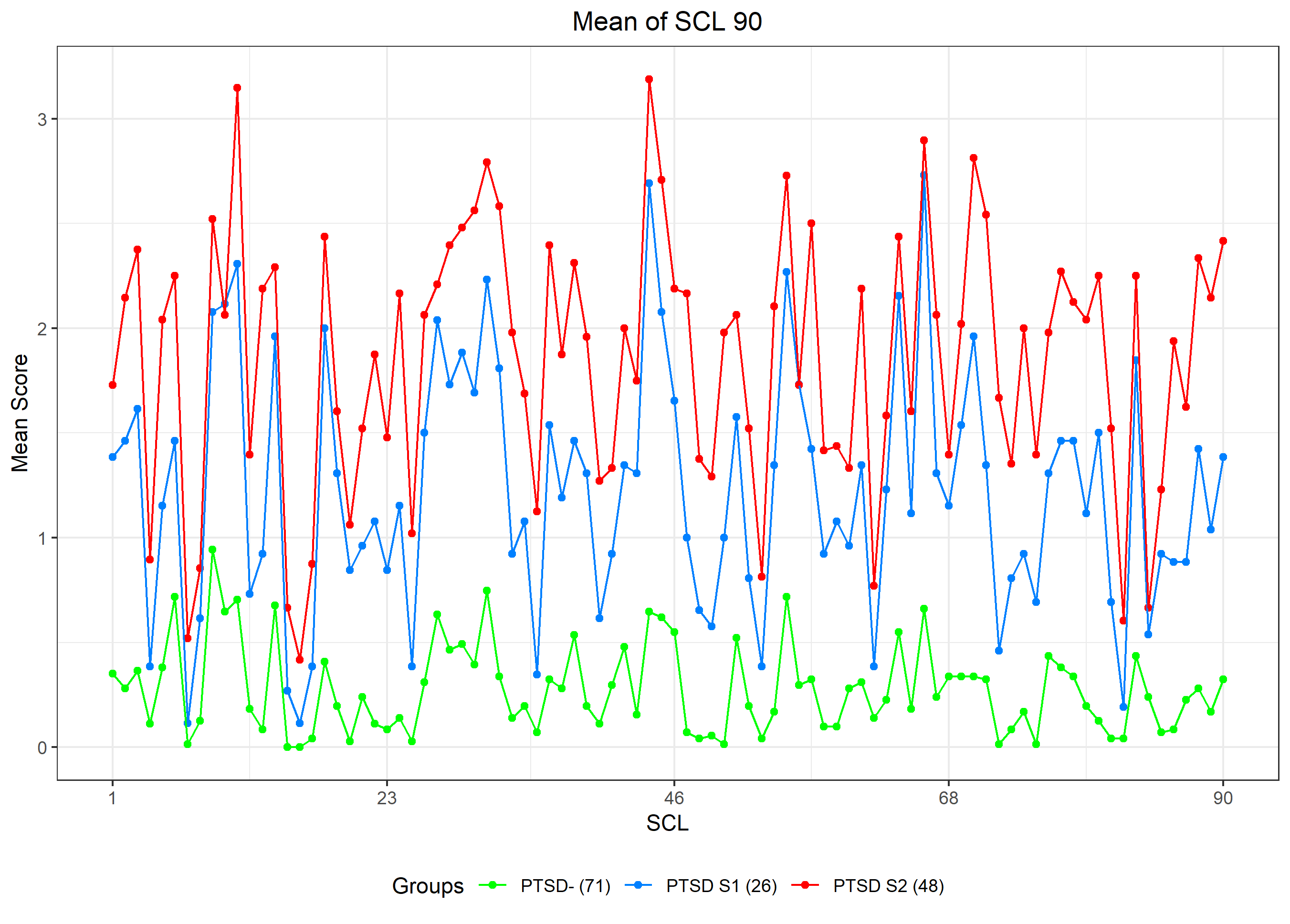
**

**
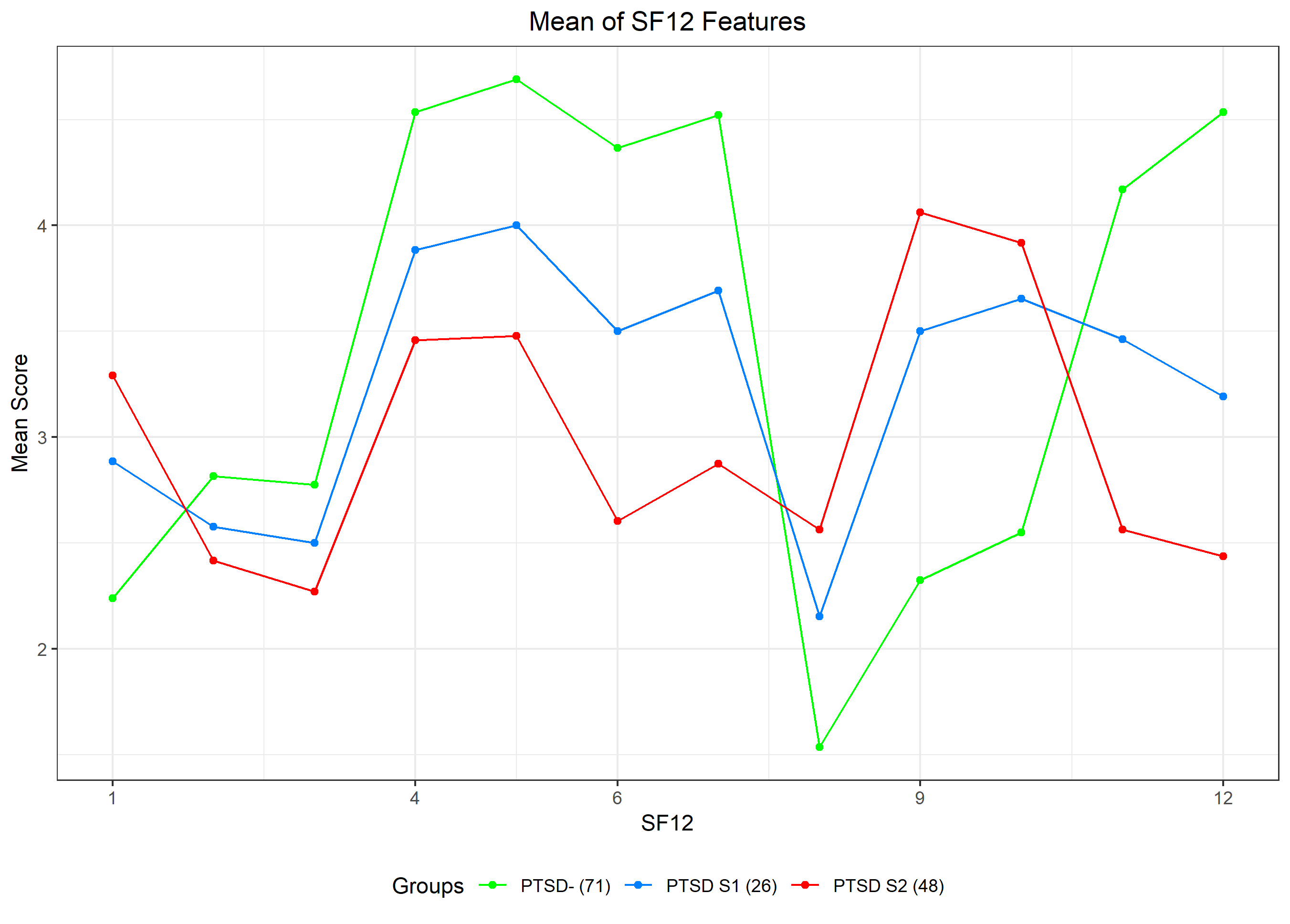
**

**
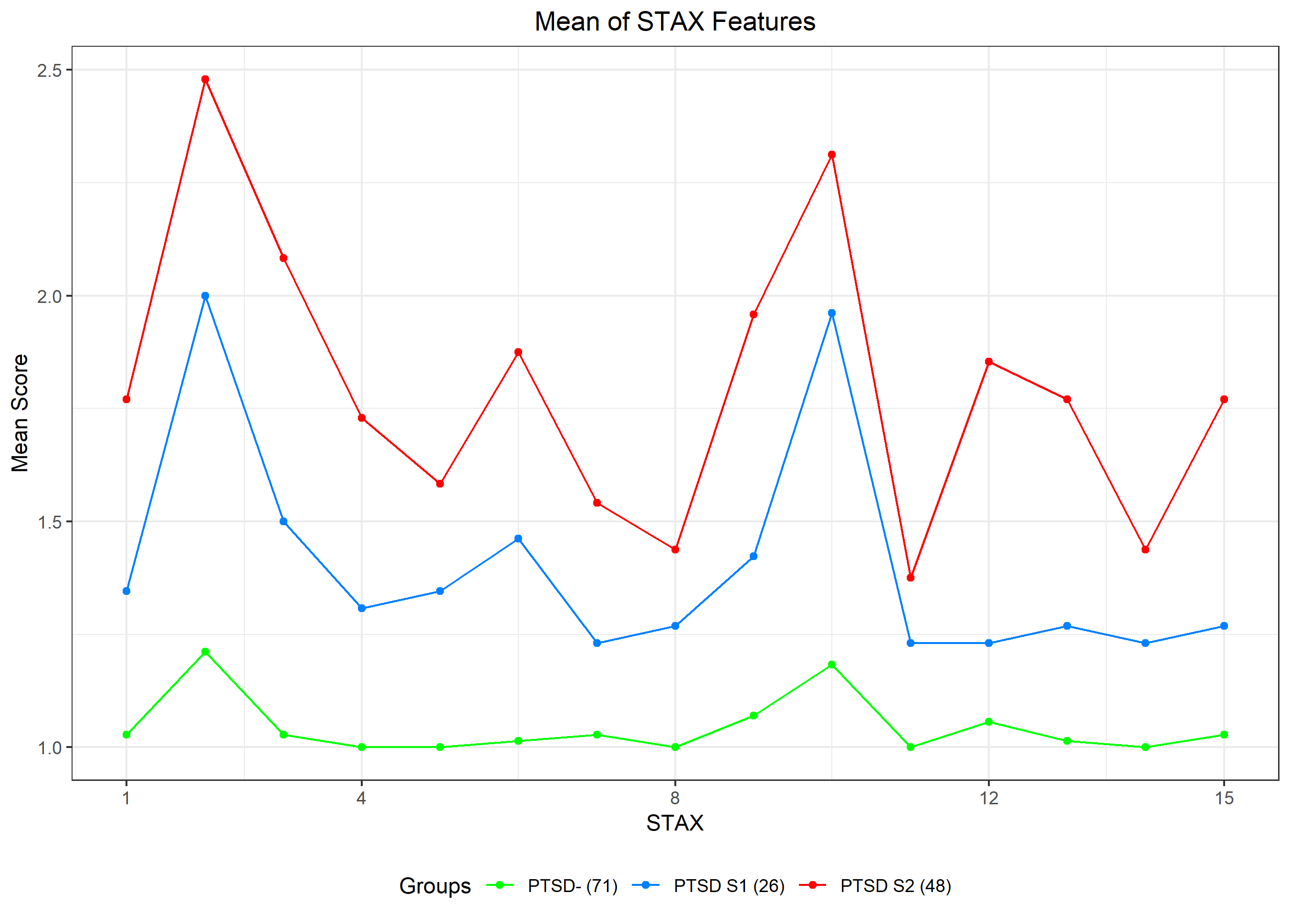
**

**Table S.4: AUCs for each feature class and all six classes combined for each intergroup comparison**

| **Prediction variable class** | | **S1 vs HCs** | | **S2 vs HCs** | **S2 vs S1** | | | **Cases vs HCs** | |
| --- | --- | --- | --- | --- | --- | --- | --- | --- | --- |
| Biological Biomarkers | | 0.911 | | 0.922 | 0.819 | | | 0.907 | |
| Current Medical Comorbidities | | 0.563 | | 0.548 | 0.545 | | | 0.537 | |
| Neuro-cognitive Functions | | 0.644 | | 0.719 | 0.549 | | | 0.667 | |
| Demographics | | 0.551 | | 0.606 | 0.552 | | | 0.569 | |
| Pre Military Trauma | | 0.571 | | 0.421 | 0.603 | | | 0.404 | |
| Psychiatric History | | 0.696 | | 0.836 | 0.500 | | | 0.813 | |
| Classes combined | | 0.931 | | 0.968 | | | 0.813 | 0.958 | |

**Table S.5: PCA Analysis: Component loadings on**

**PC1 and PC2 of subtyping total and subscale scores**

|  | PC1 | PC2 |
| --- | --- | --- |
| CAPSTOT | 0.178479 | 0.10927 |
| PCLSCORE | 0.196865 | 0.097552 |
| Sclsom | 0.181781 | -0.17822 |
| SCLOC | 0.189949 | 0.00538 |
| SCLINT | 0.190276 | 0.020687 |
| SCLDEP | 0.200061 | 0.019758 |
| SCLANX | 0.197142 | 0.047662 |
| SCLHOS | 0.188144 | -0.00253 |
| SCLPHOB | 0.188749 | -0.00289 |
| SCLPAR | 0.181071 | -0.02521 |
| SCLPSY | 0.183923 | -0.02131 |
| SCLGSI | 0.205802 | -0.01115 |
| SCLPST | 0.195926 | 0.024659 |
| SCLPSDI | 0.187852 | 0.005929 |
| PSQI | 0.164319 | -0.03199 |
| BDI_total | 0.189283 | 0.037811 |
| PANAS_PA | -0.13051 | 0.025424 |
| PANAS_NA | 0.187104 | 0.106636 |
| MCS | 0.196603 | 0.099867 |
| STAX | 0.145766 | 0.006253 |
| PSS | 0.184595 | 0.093441 |
| PDEQRV | 0.120168 | 0.304225 |
| PDEQEV | 0.165791 | 0.202939 |
| PDIrv | 0.125727 | 0.227294 |
| PDIev | 0.161135 | 0.213068 |
| ASI | 0.161358 | -0.03053 |
| sumERS | -0.13994 | -0.12447 |
| PF_T | -0.09894 | 0.437693 |
| RP_T | -0.12922 | 0.389323 |
| BP_T | -0.10867 | 0.397145 |
| GH_T | -0.10141 | 0.327599 |
| VT_T | -0.13904 | 0.047664 |
| RE_T | -0.16389 | 0.200536 |
| SF_T | -0.17326 | 0.097637 |
| MH_T | -0.16895 | -0.03379 |
| **% total variance** | **64%** | **5%** |

**Table S.6 : Subgroup Means (sds) of important biology biomarkers (n=71) identified in intergroup RFs**

| **Means (sds)** | | | |
| --- | --- | --- | --- |
|  | **Means (sds)** |  |  |
|  | **HCs** | **S1** | **S2** |
| **acthdif** | **21.96(29.02)** | **24.58(17.33)** | **27.95(19.96)** |
| **Reacthsup** | **48.23(37.66)** | **55.45(35.54)** | **63.37(37.47)** |
| **APOF.SGV** | **0.45(0.31)** | **0.54(0.28)** | **0.39(0.23)** |
| **bdnf** | **26.01(9.42)** | **30.51(7.41)** | **31.24(8.41)** |
| **cg00739770** | **-0.27(0.05)** | **-0.3(0.03)** | **-0.31(0.06)** |
| **cg01208318** | **-1.24(0.26)** | **-1.52(0.33)** | **-1.43(0.25)** |
| **cg01882498** | **-0.8(0.18)** | **-0.98(0.27)** | **-0.88(0.22)** |
| **cg03045169** | **-3.49(0.19)** | **-3.4(0.23)** | **-3.38(0.18)** |
| **cg03267026** | **-0.22(0.07)** | **-0.19(0.03)** | **-0.19(0.04)** |
| **cg04768958** | **-0.29(0.05)** | **-0.33(0.09)** | **-0.32(0.06)** |
| **cg05644921** | **-2.5(0.27)** | **-2.45(0.23)** | **-2.28(0.27)** |
| **cg06751007** | **-3.03(0.61)** | **-3.14(0.78)** | **-3.36(0.58)** |
| **cg08110688** | **-0.89(0.12)** | **-0.79(0.12)** | **-0.82(0.09)** |
| **cg11155865** | **-2.17(0.18)** | **-2.12(0.14)** | **-2(0.27)** |
| **cg12243133** | **-0.85(0.18)** | **-0.94(0.23)** | **-0.93(0.18)** |
| **cg13034868** | **-0.19(0.04)** | **-0.19(0.04)** | **-0.23(0.04)** |
| **cg13074055** | **-0.85(0.23)** | **-1.04(0.24)** | **-0.96(0.19)** |
| **cg14583127** | **-1.21(0.22)** | **-1.13(0.26)** | **-1.06(0.19)** |
| **cg14596589** | **-0.99(0.22)** | **-1.13(0.24)** | **-1.09(0.24)** |
| **cg15687973** | **-2.02(0.22)** | **-2.2(0.23)** | **-2.1(0.19)** |
| **cg16163535** | **-0.61(0.15)** | **-0.72(0.18)** | **-0.7(0.17)** |
| **cg17128892** | **-2.44(0.16)** | **-2.36(0.2)** | **-2.35(0.16)** |
| **cg18043888** | **-1.57(0.23)** | **-1.71(0.3)** | **-1.7(0.24)** |
| **cg18171204** | **-3.31(0.61)** | **-3.44(0.36)** | **-3.48(0.21)** |
| **cg18187244** | **-1.82(0.27)** | **-1.73(0.28)** | **-1.66(0.23)** |
| **cg19257562** | **-0.32(0.13)** | **-0.42(0.22)** | **-0.37(0.16)** |
| **cg19528338** | **-1.6(0.19)** | **-1.5(0.18)** | **-1.55(0.17)** |
| **cg20578780** | **-0.24(0.04)** | **-0.26(0.04)** | **-0.27(0.04)** |
| **cg20720918** | **-1.2(0.13)** | **-1.27(0.13)** | **-1.31(0.14)** |
| **cg21668832** | **-2.53(0.23)** | **-2.44(0.26)** | **-2.48(0.19)** |
| **cg23233802** | **-0.38(0.12)** | **-0.48(0.21)** | **-0.45(0.22)** |
| **cg23594345** | **-1.08(0.26)** | **-1.34(0.29)** | **-1.23(0.23)** |
| **cg24505167** | **-2.27(0.27)** | **-2.36(0.4)** | **-2.44(0.32)** |
| **cg25414209** | **-0.34(0.08)** | **-0.37(0.07)** | **-0.39(0.09)** |
| **cg26505822** | **-4.21(0.2)** | **-4.1(0.19)** | **-4.08(0.18)** |
| **Complement.C3** | **106.48(61.58)** | **155.76(77.55)** | **147.99(97.8)** |
| **dihomolinolenate203n3orn6** | **1.14(0.41)** | **0.94(0.43)** | **0.93(0.37)** |
| **elisa.npy** | **12.09(4.73)** | **13.95(6.11)** | **17.4(9.14)** |
| **eosino** | **2.65(1.54)** | **2.16(1.41)** | **2.75(1.55)** |
| **gammaglutamyltyrosine** | **1.03(0.25)** | **1.14(0.24)** | **1.19(0.34)** |
| **glucose** | **81.15(12.37)** | **85.62(27.91)** | **92.04(24.04)** |
| **hsa.let.7b.5p** | **17.82(0.7)** | **17.3(0.48)** | **17.34(0.47)** |
| **hsa.let.7g.5p** | **16.86(0.66)** | **16.33(0.62)** | **16.24(0.66)** |
| **hsa.miR.106b.3p** | **14.47(0.63)** | **14.08(0.47)** | **13.96(0.43)** |
| **hsa.miR.127.3p** | **10.8(1.83)** | **9.19(1.44)** | **9.21(1.83)** |
| **hsa.miR.1296.5p** | **9.76(1.48)** | **8.59(1.65)** | **8.31(1.98)** |
| **hsa.miR.181c.5p** | **12.8(1.55)** | **11.45(1.59)** | **11.23(2.25)** |
| **hsa.miR.186.5p** | **16.01(0.85)** | **15.34(0.87)** | **15.23(0.77)** |
| **hsa.miR.18a.5p** | **13.18(0.6)** | **12.88(0.75)** | **12.68(0.74)** |
| **hsa.miR.18b.5p** | **12.83(0.58)** | **12.56(0.73)** | **12.36(0.73)** |
| **hsa.miR.191.5p** | **16.76(1.03)** | **15.97(0.91)** | **15.8(0.97)** |
| **hsa.miR.223.5p** | **12.05(1.36)** | **11.16(1.32)** | **10.91(1.37)** |
| **hsa.miR.363.3p** | **14.86(0.4)** | **14.78(0.3)** | **14.69(0.26)** |
| **hsa.miR.370.3p** | **11.03(1.83)** | **9.45(1.5)** | **9.4(1.97)** |
| **hsa.miR.409.3p** | **13.77(1.86)** | **12.3(1.63)** | **12.45(1.99)** |
| **hsa.miR.769.5p** | **11.57(1.32)** | **10.54(1.23)** | **10.36(1.37)** |
| **hsa.miR.93.3p** | **11.05(0.97)** | **10.27(0.97)** | **10.11(0.75)** |
| **hsa.miR.93.5p** | **17.89(0.56)** | **17.45(0.53)** | **17.35(0.49)** |
| **hypoxanthine** | **1.09(0.59)** | **1.46(0.77)** | **1.53(0.74)** |
| **iminodiacetateIDA** | **1.05(0.3)** | **1.15(0.22)** | **1.12(0.29)** |
| **lactate** | **0.97(0.3)** | **1.26(0.39)** | **1.31(0.42)** |
| **mpv** | **8.99(1.07)** | **9.73(1.3)** | **9.38(1.17)** |
| **pyruvate** | **1.14(0.66)** | **1.82(1.27)** | **1.48(0.84)** |
| **tyrosine** | **1.01(0.19)** | **1.13(0.28)** | **1.17(0.31)** |
| **X30683756** | **5.99(2.64)** | **7.51(2.46)** | **7.21(2.57)** |
| **X45411864** | **9.59(2.78)** | **8.77(3.34)** | **8.23(3.52)** |
| **X47209677** | **-1.22(1.94)** | **-0.41(1.13)** | **-0.51(1.51)** |
| **X49372131** | **-4.65(2.91)** | **-5.9(2.94)** | **-5.59(3.17)** |
| **X6707521** | **7.48(3.24)** | **8.88(2.84)** | **8.11(2.84)** |
| **X70672983** | **-4.63(3.12)** | **-5.02(3.23)** | **-5.66(2.84)** |
| **X75938326** | **-2.36(2.52)** | **-1.71(2.15)** | **-1.3(1.48)** |

Change >.04  and marker  comparison of S2 with S1 Red = increasing ;  Green= decreasing; Yellow = change <.04

**Table S.7: Mean (sds) values of variables entering RF in feature classes of**

**Neuro-cognition and past psychiatric diagnoses**

| **Means (sds)** |  |  |  |
| --- | --- | --- | --- |
|  | **HCs** | **S1** | **S2** |
| **Neurocognition** |  |  |  |
| FAS: Initial Letter Verbal Fluency Test | 43.38 (10.37) | 40.27 (10.92) | 36.94 (10.30) |
| Vocabulary (ranked 1) | 41.75 (8.75) | 35.27 (8.34) | 35.83 (9.46) |
| Speed Coding | 9.97 (2.59) | 9.77 (2.21) | 8.10 (2.41) |
| Visual Memory | 20.87 (4.80) | 19.50 (4.12) | 18.25 (4.85) |
| Digit Span | 10.31 (3.21) | 9.08 (2.21) | 8.69 (2.91) |
| Spatial Memory | 10.34 (3.05) | 9.96 (2.24) | 9.04 (3.15) |
| **SCID Lifetime psychiatric diagnoses (number, %)** |  |  |  |
| Major depression (ranked 1) | 16 (22.54%) | 18 (69.2%) | 45 (93.75%) |
| Phobias | 4 (5.63%) | 3 (11.54) | 4 (8.33%) |
| Generalized anxiety disorder | 0 (0.00%) | 2 (7.69%) | 4 (8.33%) |
| Anxiety NOS | 0 (0.00%) | 1 (3.85%) | 4 (8.33%) |
